# Integrative profiling of gut microbiome, bacteriophagenome, and predicted metabolome in obese adults: novel insights into intervention targets

**DOI:** 10.1186/s12866-025-04682-1

**Published:** 2026-01-29

**Authors:** Lu Li, Huan Wang, Yuan Gao, Bei Zhang, Yongfu Chen

**Affiliations:** 1 Key Laboratory of Dairy Biotechnology and Engineering, Ministry of Education, Inner Mongolia Agricultural University, Hohhot, China; 2 Key Laboratory of Dairy Products Processing, Ministry of Agriculture and Rural Affairs, Inner Mongolia Agricultural University, Hohhot, China; 3 Inner Mongolia Key Laboratory of Dairy Biotechnology and Engineering, Inner Mongolia Agricultural University, Hohhot, China; 4 Department of Clinical Nutrition, Inner Mongolia People’s Hospital, Hohhot, China; 5 College of Food Science and Engineering, Inner Mongolia Agricultural University, 306 Zhaowuda Road, Inner Mongolia Autonomous Region, Hohhot, People’s Republic of China

**Keywords:** Obese adults, Body composition, Gut microbiome, Bacteriophages, Gut metabolic modules, Gut predicted metabolites

## Abstract

**Background:**

Gut microecology-targeted intervention shows significant potential in correcting metabolic imbalances associated with the global obesity epidemic. While the gut microbiome in obesity has been widely studied, prior work has largely examined individual microbial or metabolic dimensions in isolation. Thus, this study aims to systematically characterize obesity-associated gut microbial features through a multidimensional integrated analysis that jointly considers gut microbiome, bacteriophagenome, predicted metabolome, and body composition traits.

**Results:**

Body composition parameters (body mass index [BMI], body fat rate [BFR], waist-to-hip ratio [WHR], muscle mass-to-body weight ratio [MM/BW], and basal metabolic rate-to-fat-free mass ratio [BMR/FFM]) along with species-level gut microbiota (SGBs), bacteriophages, gut metabolic modules (GMMs), and gut predicted metabolites (GPMs) were compared between obese adults (OB group, *n* = 36) and healthy adults (HE group, *n* = 36) to construct a multidimensional association network. The OB group showed significantly higher BMI, BFR, and WHR (*P* < 0.05), while MM/BW and BMR/FFM were reduced (*P* < 0.05). The omics analysis identified 21 key SGBs (including *Faecalibacillus intestinalis, Blautia_*A *wexlerae, Blautia_*A sp900066335*, Anaerostipes amylophilus, Anaerobutyricum hallii, Dorea formicigenerans*, etc.), two bacteriophages (Myoviridae, Inoviridae), 16 GMMs (including glycine degradation, methionine degradation I, aspartate degradation I, etc.), and 16 GPMs (including N-acetylspermidine, N-acetylhistidine, imidazole propionate, etc.) that were significantly altered in the OB group (*P* < 0.05). Correlation network analysis revealed that these differential features were associated with body composition indicators through multi-level and distinct relationships (*P* < 0.05, |r|≥ 0.4), suggesting their potential relevance as intervention targets.

**Conclusion:**

This study provides hypothesis-generating insights into gut ecosystem-based alterations associated with obesity, and these distinctive gut-related features warrant validation as potential biomarkers in prospective studies.

**Supplementary Information:**

The online version contains supplementary material available at. 10.1186/s12866-025-04682-1.

## Introduction

Obesity has emerged as a global epidemic and one of the most pressing public health challenges. In 2021, elevated body mass index (BMI) was estimated to account for 3.7 million deaths worldwide, primarily from cardiovascular disease, diabetes, cancer, neurological disorders, and other related conditions [1]. By 2025, the number of overweight and obese adults is projected to reach 3.8 billion, further intensifying the global health and economic burden of obesity [2]. Addressing this crisis requires urgent, sustained, and targeted strategies for effective management. Although dietary habits, lifestyle factors, and genetic predisposition are well-recognized risk contributors, conventional interventions often encounter limitations in achieving long-term weight control and preventing obesity-related complications, primarily due to insufficient impact on underlying metabolic regulation and poor sustainability [3]. Recently, increasing evidence has highlighted the role of gut microbiota in modulating host energy metabolism and fat accumulation through multiple mechanisms, positioning it as a central focus in obesity research [4, 5].

Obesity and gut microbiota are interconnected through a complex and bidirectional relationship. Evidence from multiple studies demonstrates that, compared with healthy individuals, obese individuals typically represent reduced microbial diversity and altered proportions of dominant taxa [6–9]. For example, the relative abundance of Bacteroidetes is frequently decreased, whereas that of Bacillota is often increased, although considerable heterogeneity has been reported across studies, primarily reflecting individual variability [10–12]. Studies have shown that alterations in gut microbial composition and structure are closely linked to host metabolic health and energy balance [13–15]. Moreover, a placebo-controlled trial reported that oral administration of fecal microbiota transplantation (FMT) capsules from lean donors to obese individuals induced significant shifts in gut microbial communities and bile acid profiles, making them more similar to those observed in lean donors. This provides additional evidence for the central role of gut microbiota in obesity [16]. However, studies that focus exclusively on bacterial communities often fail to capture the full complexity of the gut ecosystem. Bacteriophages, as key regulators of bacterial populations, have increasingly attracted attention. A previous study demonstrated that FMT from lean donors administered to obese individuals also led to significant shifts in gut bacteriophages populations [17]. Evidence suggests that bacteriophages modulate bacterial abundance, community composition, functional capacity, and metabolite production by lysing host bacteria or facilitating horizontal gene transfer, indirectly contributing to host metabolic regulation [18–22]. For example, specific bacteriophages have been shown to influence bacterial metabolic pathways, therefore affecting key metabolites that play essential roles in maintaining host energy homeostasis [18]. Despite growing interest, research on obesity and the gut bacteriophages community remains in its early stages, and the potential network-level interactions between bacteriophages and host metabolism are not yet fully understood. In parallel, predictive metabolomics, which infers metabolic profiles from microbial genomic pathways, provides insights into gut microbe-derived metabolites such as amino acids, bile acids, and nucleosides. These metabolites directly affect host physiological functions and act as essential mediators, linking the microbiome to host metabolism [23]. Gut bacteria, bacteriophages, and metabolites form a highly interconnected regulatory network. Although previous studies have examined the individual contributions of these components in obesity, most remain limited to single-omics analyses. This narrow focus hampers the systematic identification of synergistic targets within the microbial ecosystem. Significant inter-individual variability also highlights the need for comprehensive, longitudinal investigations to pinpoint key regulatory nodes. Therefore, constructing an integrated bacteria–bacteriophage–metabolite network and identifying novel functional targets represent important directions with significant scientific and clinical implications for the prevention and management of obesity.

This study aims to establish multi-level association networks and identify potential intervention targets by systematically analyzing body composition, gut microbiota, bacteriophages, gut metabolic modules, and predictive metabolites in obese adults, generating new scientific evidence to inform clinical practice. Beyond enriching the research framework of gut microbiology and metabolomics in obesity, this work increases understanding of the complex biological mechanisms driving the disease. Furthermore, it provides a theoretical basis and potential targets for future personalized nutritional strategies, as well as gut microecology-based interventions such as probiotic, postbiotic or bacteriophage, ultimately supporting the advancement of precision medicine in obesity management.

## Methods

### Study design and recruitment

**Fig. 1:**
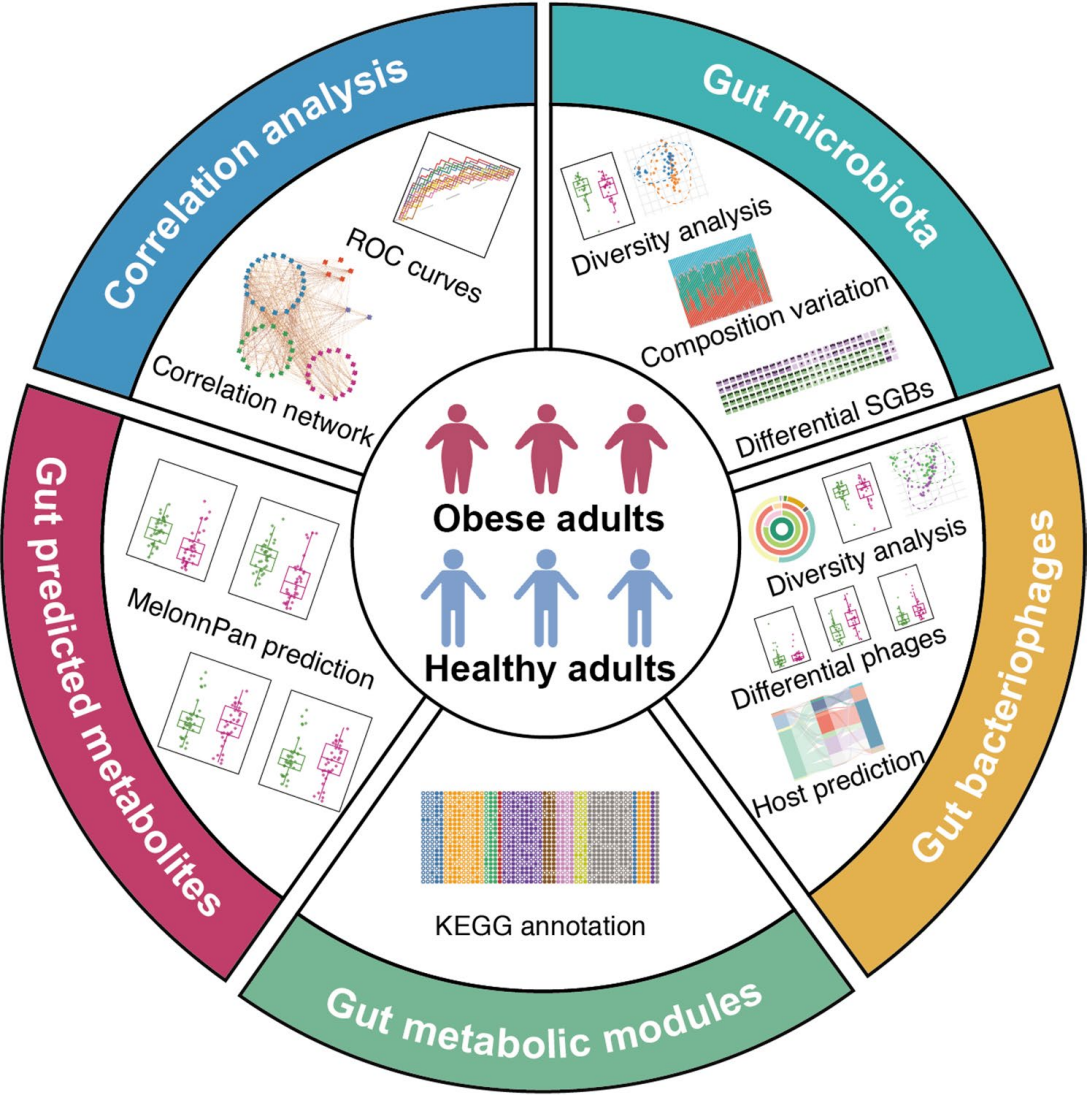
Schematic diagram of the study. This study systematically investigated differences in body composition, gut microbiota, gut bacteriophages, gut metabolic modules, and predicted gut metabolites between obese and healthy adults to identify potential targets for obesity. Finally, a correlation network was constructed to elucidate the interrelationships among these multi-omics datasets

This cross-sectional study aimed to characterize the gut microbiome, bacteriophages, gut metabolic modules, and predicted metabolites in obese and healthy adults, to identify potential novel targets for obesity interventions (Fig. 1). Eligible participants were adults aged 18–65 years, of either sex, with a BMI of 18.5–24.0 kg/m^2^ or ≥ 28.0 kg/m^2^, and able to comply with study procedures, including body composition measurements and fecal sample collection. Exclusion criteria included pregnancy or lactation; a history of diabetes, hypertension, severe gastrointestinal or hematologic disorders; impaired hepatic, renal, or cardiac function; and use of antibiotics, anti-obesity medications, probiotics, prebiotics, or postbiotics within the previous month.

A total of 72 participants were enrolled in the study. Based on the Chinese adult BMI classification, individuals were categorized into the obese group (OB, *n* = 36, BMI ≥ 28.0 kg/m^2^) and the healthy group (HE, *n* = 36, 18.5 ≤ BMI < 24.0 kg/m^2^) [24]. At baseline, body composition was assessed, and fecal samples were collected in tubes containing protective solution. These samples were then subjected to high-throughput metagenomic sequencing, enabling the comprehensive characterization of the gut microbiome, bacteriophages, gut metabolic modules (GMMs), and gut predicted metabolites (GPMs).

### Measurement of body composition

Body composition was evaluated using a standing, eight-electrode, multi-frequency bioelectrical impedance analysis (BIA) device (BCA-1C, Tsinghua Tongfang Co., Ltd., Beijing, China) [25]. This system measures whole-body impedance and, based on sex, age, height, and body weight (BW), automatically calculates BMI, fat mass (FM), muscle mass (MM), waist-to-hip ratio (WHR), and basal metabolic rate (BMR) through its embedded algorithm. Other indicators were derived, including body fat rate (BFR; FM/BW × 100%), relative MM (MM/BW × 100%), and BMR relative to fat-free mass (FFM; BMR/FFM × 100%), to assess the proportion of muscle and metabolic capacity of FFM. All measurements were performed in strict compliance with the manufacturer's protocol. To ensure accuracy and data comparability, participants were instructed to fast or remain at least 2–3 h postprandial, empty their bladders, and avoid vigorous exercise or excessive fluid intake before testing.

### Metagenomic sequencing, binning, and taxonomic annotation

The DNA from fecal samples was extracted using the SPINeasy DNA Kit for Feces (MP Biomedicals, Santa Ana, CA, USA) [26]. Library preparation was performed using the MGIEasy FS DNA Prep Set (MGI Tech Co., Ltd., Shenzhen, China) following standard protocols. Metagenomic sequencing was conducted on the MGISEQ-2000 platform (MGI Tech Co., Ltd., Shenzhen, China) to generate 150 bp paired-end reads. Quality control of raw metagenomic reads was carried out using the KneadData pipeline (http://huttenhower.sph.harvard.edu/kneaddata), and Bowtie2 was applied to remove reads contaminated with human DNA. The resulting clean data were retained for further analyses.

Clean reads from each sample were assembled into contigs using MEGAHIT [27]. Contigs larger than 2,000 bp were selected for binning using MetaBAT2, DAS Tool, and VAMB with default parameters [28–30]. Metagenome-assembled genomes (MAGs) were obtained by combining all the bins. Then, BWA-MEM2 was applied to align the reads with corresponding contigs, and contig depth was measured by Samtools and jgi_summarize_bam_contig_depths [31]. The completeness and contamination of MAGs were assessed using CheckM, and high-quality MAGs (completeness ≥ 80%, contamination ≤ 5%) were then clustered. The most representative genomes from each cluster were then selected to generate species-level genome bins (SGBs) using dRep with the parameters -pa 0.95 and -sa 0.95 [32, 33].

SGBs were annotated using Kraken2 in combination with the NCBI non-redundant Nucleotide Sequence Database [34]. Prodigal was applied to predict putative genes in the contigs [35]. The predicted genes were searched against the UniProt Knowledgebase (UniProtKB, version 2020) using DIAMOND's blastp with default settings. The relative abundance of each SGB was assessed using CoverM (https://github.com/wwood/CoverM) with the options "–minread-percent-identity 0.95 –min-covered-fraction 0.4".

### Bacteriophagenome and host identification

Interactions between bacteriophages and their bacterial hosts were investigated using VIBRANT for bacteriophage identification and host association based on contigs from metagenomic assembly. Potential bacteriophages were first detected in contigs larger than 1,000 bp using VIBRANT with default parameters. [36]. All contigs were then evaluated with CheckV, and CD-HIT (https://github.com/weizhongli/cdhit) was applied to identify viral contigs exceeding 5,000 bp [37, 38]. Clustering was performed using a 95% nucleotide identity threshold, requiring at least 80% of sequences within a cluster to meet this criterion, resulting in viral operational taxonomic units (vOTUs). To assess novelty, vOTUs were compared against viral genomes in the Metagenomic Gut Virus catalog (accessed July 2021) [39]. Finally, the relative abundance of vOTUs was calculated using the CoverM-contig pipeline (https://github.com/wwood/CoverM) with the following parameters: –min-read-percent-identity 0.95, –min-read-aligned-percent 0.5, –proper-pairs-only, and –exclude-supplementary.

### Prediction of GMMs and GPMs

A module-based analytical framework, combined with the MetaCyc metabolic database, was used to predict GMMs encoded by SGBs [40]. Predicted open reading frames were aligned with the Kyoto Encyclopedia of Genes and Genomes (KEGG) Orthology database to annotate key metabolic modules for each SGB. OmixerRPM was then applied to identify SGBs encoding the corresponding modules, using a cutoff setting of –c 0.66 [41].

GPMs were predicted based on high-quality sequences. One million reads per sample were subsampled using seqtk (https://github.com/lh3/seqtk), and the subsampled reads were compared by the blastx function of DIAMON "-query-cover 90-id 50". The best hit for each gene was selected for calculating the gene abundance profile of each sample. Then the MelonnPan-predict workflow was used to convert gene abundances into a predicted metabolomic table [23]. It should be noted that GPMs generated by MelonnPan represent computationally inferred metabolite profiles with inherent uncertainty rather than experimentally measured metabolites.

### Statistical analysis

Statistical analysis and graphical visualization were performed using R (v.4.5.0) and Adobe Illustrator. The R packages (e.g., vegan, optparse, mixOmics, ggplot2, and ggpubr) were used to calculate the Shannon index and to execute principal coordinate analysis (PCoA, Bray–Curtis distance), the Adosim test (999 permutations), and Procrustes analysis. Pearson correlation analysis was conducted to compare the Shannon index between SGBs and bacteriophages. Correlation network analysis was employed between SGBs, bacteriophages, GMMs, GPMs, and body composition indicators, using a cut-off of |r|≥ 0.4 based on Spearman correlation coefficients. The Wilcoxon rank-sum test was used to assess differences in variables between groups. *P* values were adjusted using the Benjamini–Hochberg procedure, and *P* < 0.05 was considered statistically significant.

## Results

### Baseline characteristics of study participants

In this study, body composition was assessed in a total of 72 participants, including 36 individuals in the obese group (OB; 18 males and 18 females) and 36 in the healthy group (HE; 12 males and 24 females) (Table 1). No significant difference was observed in the sex distribution between the two groups (Chi-square test, *P* = 0.232). The mean age was 31.22 ± 4.01 years in the OB group and 29.58 ± 4.58 years in the HE group, with no significant difference (*P* = 0.090). BW, BMI, FM, BFR, MM, WHR, and BMR were all significantly higher in the OB group compared with the HE group (*P* = 4.61 × 10⁻^13^, *P* = 3.02 × 10⁻^13^, *P* = 3.03 × 10⁻^13^, *P* = 4.72 × 10⁻⁹, *P* = 2.28 × 10⁻⁷, *P* = 2.88 × 10⁻^13^, *P* = 2.06 × 10⁻⁶, respectively). In comparison, MM/BW and BMR/FFM were significantly lower in the OB group than in the HE group (*P* = 4.69 × 10⁻⁸ and *P* = 1.02 × 10⁻⁹, respectively). These findings indicated that the two groups were comparable in demographic characteristics but differed significantly in body composition index, providing a basis for further comparative analyses of the gut microbiome, bacteriophages, and predicted metabolome.

**Table 1:** Baseline characteristics of study participants

Characteristics	Obese adults (*n* = 36)	Healthy adults (*n* = 36)	*P* value
Gender (male/female)	18/18	12/24	0.232
Age (years)	31.22 ± 4.01	29.58 ± 4.58	0.090
Height (m)	1.71 ± 0.10	1.67 ± 0.08	0.072
Weight (kg)	95.23 ± 17.00	59.83 ± 8.08	4.61e-13
Body mass index (kg/m^2^)	32.33 ± 3.61	21.50 ± 1.65	3.02e-13
Body fat mass (kg)	32.80 ± 5.71	14.24 ± 3.50	3.03e-13
Body fat rate (%)	34.92 ± 5.71	24.06 ± 5.92	4.72e-09
Muscle mass (kg)	58.80 ± 14.03	42.57 ± 7.76	2.28e-07
Muscle mass/weight (%)	61.25 ± 5.80	70.89 ± 5.63	4.69e-08
Waist/hip	1.02 ± 0.06	0.85 ± 0.03	2.88e-13
Basal metabolic rate (kcal/day)	1892.48 ± 413.24	1506.67 ± 218.39	2.06e-06
Basal metabolic rate/fat free mass (kcal/day/kg)	30.44 ± 1.31	33.26 ± 1.53	1.02e-09

### Alterations in gut microbiota in obese adults

**Fig. 2:**
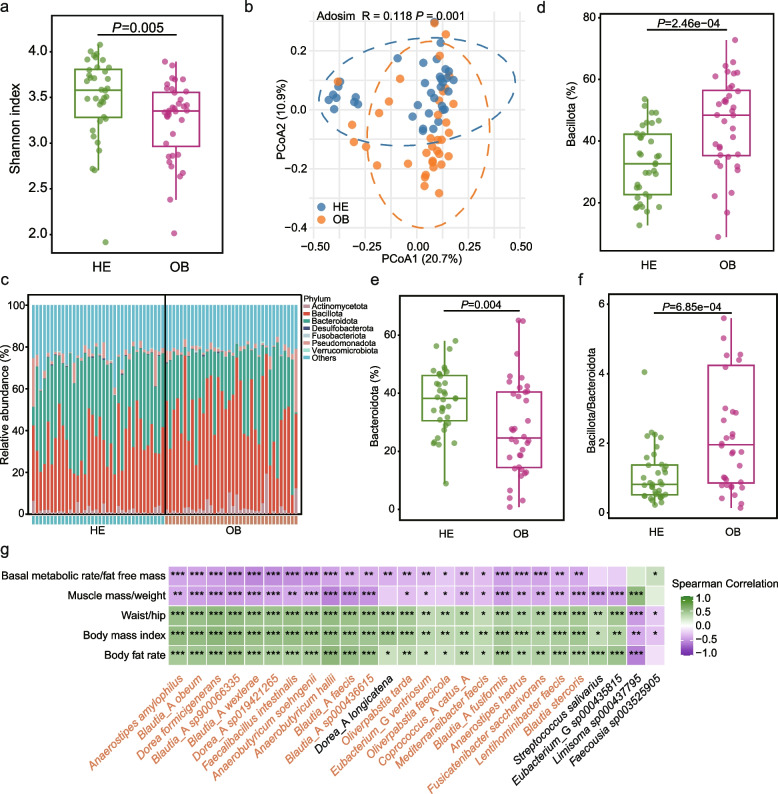
Changes in gut microbiota and correlation between SGBs and body composition indicators in obese adults. **a** Shannon diversity index. **b** Principal coordinates analysis (PCoA; Bray–Curtis distance) score plots with Adosim test. **c** Composition and relative abundance of gut microbiota at the phylum level. Different colors represent different phyla; the color corresponding to each bar segment indicates the relative abundance of the respective phylum. **d-f** Differential relative abundances of Bacillota, Bacteroidota, and the Bacillota/Bacteroidota ratio between healthy and obese adults. The central line in each box plot denotes the median, the box edges represent the interquartile range, and the whiskers extend to values outside the upper and lower quartiles. Group differences were assessed using the Wilcoxon test. **e** Spearman’s correlation coefficients between differential SGBs and body mass index, body fat rate, waist-to-hip ratio, muscle mass-to-body weight ratio, and basal metabolic rate-to-fat-free mass ratio. Color intensity indicates the strength of the correlation. *P* values were corrected using the Benjamini–Hochberg procedure. *P < 0.05, **P < 0.01, ***P < 0.001

To examine the differences in gut microbiota structure and composition between obese and healthy adults, the Shannon index was calculated to evaluate microbial richness and evenness, with higher values indicating greater species diversity and a more balanced distribution. The Shannon index was significantly lower in the OB group compared to the HE group (OB: 3.25 ± 0.42 *vs.* HE: 3.50 ± 0.45, *P* = 0.005), indicating reduced species richness and evenness under obese conditions (Fig. 2a). β-diversity analysis using Bray–Curtis distance and PCoA further revealed a clear separation between the OB and HE groups, with significant differences in community structure (Adosim, *R* = 0.118, *P* = 0.001), suggesting that obesity not only decreases gut microbial diversity but also reshapes the overall community architecture (Fig. 2b). These results indicate that the gut microbiota of obese adults shows reduced diversity and structural alterations, which may contribute to the metabolic dysregulation associated with obesity; however, these alterations represent associations and do not establish causality.

A comparative analysis was performed on the gut microbiota composition between the groups. Results indicated that the dominant phyla (relative abundance > 1%) in both groups were the Bacillota, Bacteroidota, Actinomycota, and Pseudomonadota*.* Among these, the Bacillota (OB: 45.45 ± 14.91%; HE: 32.94 ± 11.45%) and Bacteroidetes (OB: 27.82 ± 16.46%; HE: 37.54 ± 10.85%) were the predominant phyla in both groups, though their relative abundances showed significant differences (Fig. 2c). Compared with healthy adults, obese adults showed a significantly increased relative abundance of the Bacillota (*P* = 2.46 × 10^–4^) and a significantly decreased relative abundance of the Bacteroidetes (*P* = 0.004), leading to a significantly elevated Bacillota/Bacteroidetes ratio (*P* = 6.85 × 10^–4^) (Fig. 2d-f). This indicated alterations in the composition of the gut microbiota in obese adults.

To further investigate species-level differences, a systematic comparison of the gut microbiota was performed between the groups. Among the 263 identified SGBs, 89 SGBs showed significant differences between the two groups (*P* < 0.05). To identify key microbial communities closely associated with obesity, fold change (FC) and variable importance in projection (VIP) metrics were combined for screening. This yielded 26 significantly differentially abundant SGBs (*P* < 0.05, 2 ≥ FC ≥ 0.5, VIP > 1.5; Table S1, Fig. 2g). Among these, *Limisoma* sp000437795 and *Faecousia* sp003525905 showed significantly reduced relative abundance in the OB group (*P* < 0.05), whereas the remaining 24 SGBs showed significant increases in the OB group (*P* < 0.05). Correlation analysis further revealed relationships between these differential SGBs and body composition metrics. Correlation analysis revealed that 21 of the 26 differentially abundant SGBs were positively associated (*P* < 0.05) with BMI, BFR, and WHR, and negatively associated with MM/BW and BMR/FFM (Fig. 2g). Integrating these results, 21 key differential SGBs were identified as being closely linked to body composition, indicating that they may serve as potential targets for further validation in obesity research.

### Changes in gut bacteriophageome in obese adults

**Fig. 3:**
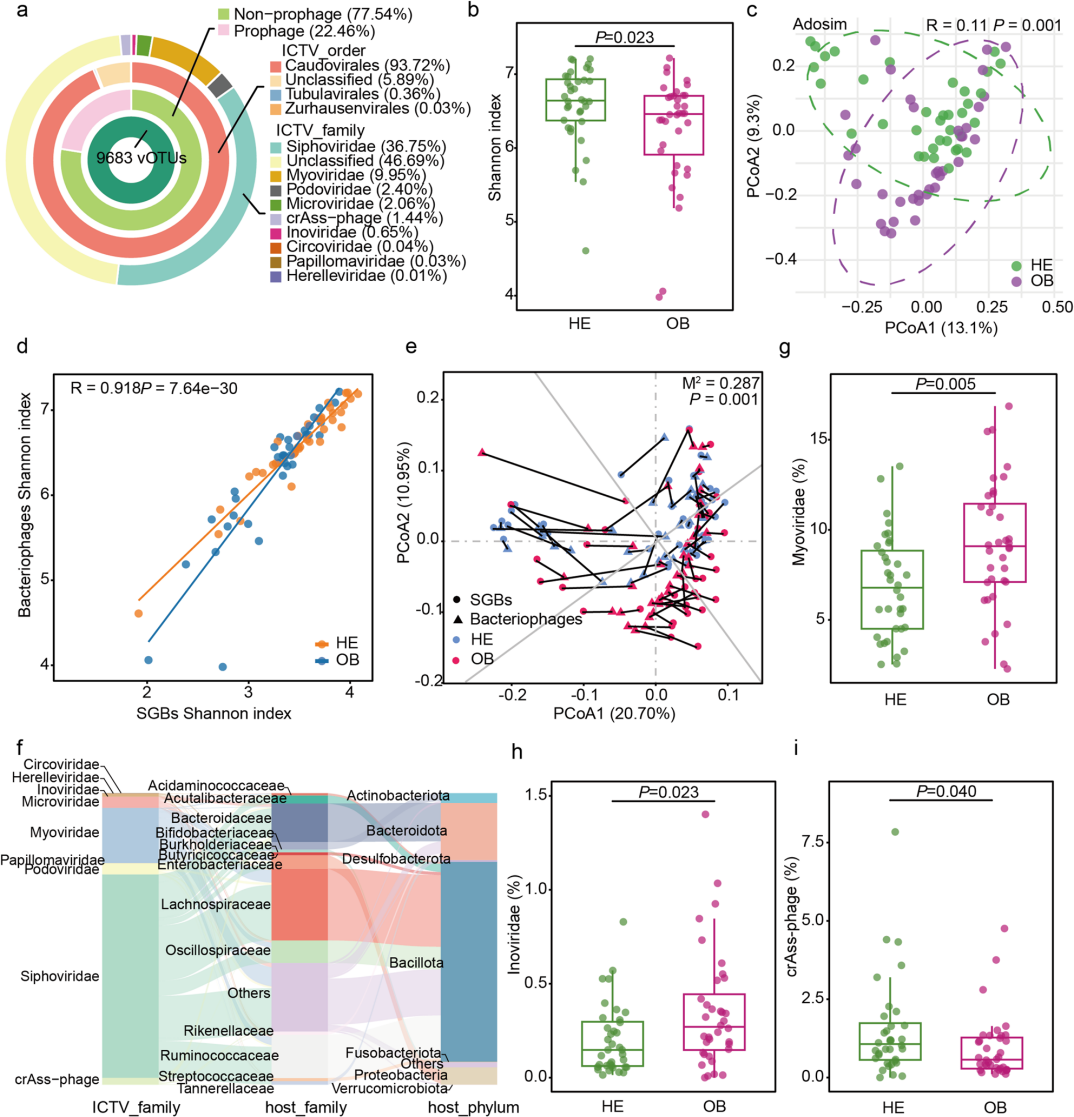
Changes in gut bacteriophages between healthy and obese adults.** a** Circular diagram showing whether each annotated viral operational taxonomic unit (vOTU; *n* = 9,683) is classified as “prophage” or “non-prophage,” along with its order- and family-level taxonomic distributions according to the International Committee on Taxonomy of Viruses. **b** Shannon index. **c** Principal coordinates analysis (PCoA; Bray–Curtis distance) score plots with Adosim test. **d** Pearson correlation between bacterial and bacteriophages Shannon index. **e** Procrustes analysis comparing SGBs and bacteriophages between groups. **f** Family-level classification of bacteriophages and their corresponding bacterial hosts at both the family and phylum levels. **g-i** Significantly different bacteriophages between the two groups. The central line in each box plot denotes the median; the box edges represent the interquartile range, and the whiskers extend to values outside the upper and lower quartiles. Differences between groups were assessed using the Wilcoxon test, and *P* < 0.05 considered statistically significant

Considering the role of bacteriophages in reshaping the gut microbiota, changes in diversity and composition of the phage community in the intestines of obese adults were investigated. By aligning the dataset against the metagenomic phage catalogue, 9,683 non-redundant bacteriophages operational taxonomic units were annotated. These comprised 2,175 prophages and 7,508 non-prophages (Fig. 3a). Taxonomic annotation of these sequences revealed high occurrence rates for Siphoviridae (36.75%), Myoviridae (9.95%), Podoviridae (2.40%), Microviridae (2.06%), and crAss-phage (1.44%) were prevalent (> 1% abundance), all belonging to the Caudovirales order (93.72%) (Fig. 3a).

Diversity analysis revealed that the bacteriophage Shannon index in the OB group was significantly lower than that in the HE group (*P* = 0.023), indicating reduced bacteriophage diversity and evenness in the gut phageome of obese adults (Fig. 3b). β-diversity analysis using Bray–Curtis distance and PCoA demonstrated a clear separation in community structure between the two groups (Adosim, *P* = 0.001, R = 0.11), reflecting significant alterations in the gut phageome under obesity (Fig. 3c). Correlation analysis between bacteriophages and SGBs revealed a strong positive relationship between the bacteriophage Shannon index and the SGB Shannon index (Pearson, *R* = 0.918, *P* = 7.64 × 10⁻^3^⁰) (Fig. 3d). Procrustes analysis further indicated significant concordance between their community structures (M^2^ = 0.287, *P* = 0.001), suggesting robust interactions between bacteriophages and gut bacterial communities (Fig. 3e).

The distribution of bacteriophage sequences within bacterial host genomes was further investigated. Of the 9,683 vOTUs, computational host prediction identified 8,934 vOTUs with putative bacterial associations, including 7,760 vOTUs linked to known host bacterial families; these predicted associations require experimental validation (Fig. 3f). Siphovirida, the most prevalent intestinal bacteriophage family, primarily parasitises hosts within the Bacillota and Bacteroidota*,* predominantly including the Lachnospiraceae, Ruminococcaceae, Bacteroidaceae, Oscillospiraceae, and Acutalibacteraceae. Myoviridae and Podoviridae predominantly infect hosts within Bacillota, Proteobacteria, and Bacteroidota, primarily including the Lachnospiraceae, Ruminococcaceae, Enterobacteriaceae, Oscillospiraceae, and Burkholderiaceae. Microviridae mainly infect hosts within the Bacteroidaceae and Oscillospiraceae. crAss-phage predominantly infects hosts within the Bacteroidaceae. These findings suggested a close interaction between intestinal bacteriophages and their bacterial hosts.

Differential abundance analysis revealed that the relative abundances of Myoviridae (*P* = 0.005) and Inoviridae (*P* = 0.023) were significantly higher in the OB group compared to the HE group, whereas crAss-phage was significantly lower in the OB group (*P* = 0.040) (Fig. 3g–i). This suggests that obesity may be linked to the expansion of specific bacteriophage communities and corresponding shifts in ecological niches.

### Differences in GMMs and GPMs in obese adults

**Fig. 4:**
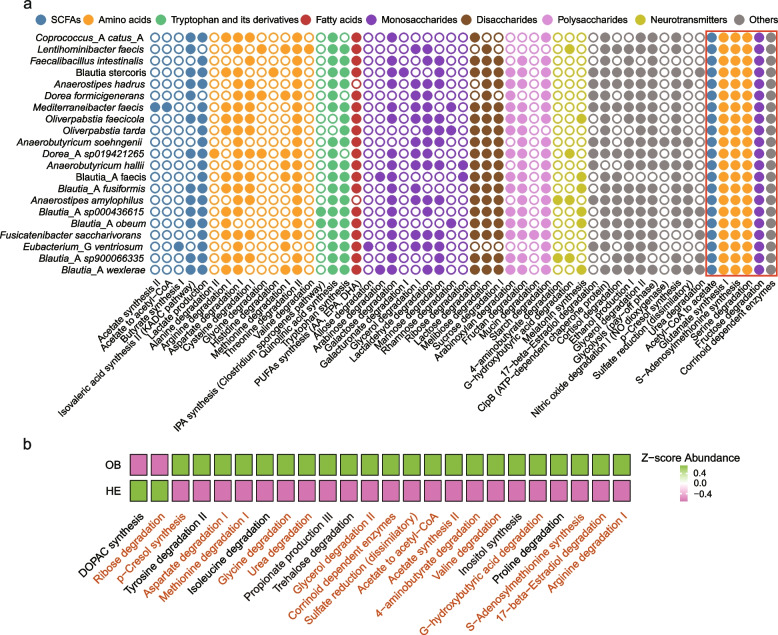
Changes in GMMs between health and obese adults.** a** Distinction of 53 GMMs belonging to 9 metabolic modules, including short-chain fatty acids, amino acids, tryptophan and its derivatives, fatty acids, monosaccharides, disaccharides, polysaccharides, neurotransmitters, and other metabolic modules across 21 differential SGBs. Red boxes highlight the six GMMs commonly enriched across these 21 SGBs. **b** Differential GMMs between HE and OB groups. Colors indicate the Z-score of the relative abundance of each GMM within each group. GMMs labeled in orange represent differential GMMs enriched in differential SGBs

Differences in gut microbiota and bacteriophage communities fundamentally influence intestinal metabolic characteristics. A genome-centric metabolic reconstruction model was constructed using the MetaCyc and KEGG databases to analyze obesity-induced alterations in GMMs systematically. The 21 previously identified key differentially expressed SGBs encoded a total of 53 GMMs, covering nine metabolite categories: short-chain fatty acids (SCFAs), amino acids, tryptophan derivatives, fatty acids, monosaccharides, disaccharides, polysaccharides, neurotransmitters, and other metabolic modules (Fig. 4a). The six metabolic modules were encoded by all differential SGBs: acetyl-CoA to acetate, glutamate synthesis I, S-adenosylmethionine synthesis, serine degradation, fructose degradation, and corrinoid-dependent enzymes. Inter-group differential analysis further identified 24 significantly altered GMMs. Among these, ribose degradation and 3,4-Dihydroxyphenylacetic acid (DOPAC) synthesis were significantly reduced in the OB group compared to the HE group (*P* < 0.05). At the same time, the remaining 22 GMMs were significantly elevated in the OB group (*P* < 0.05) (Fig. 4b, Table S2). Significantly, 17 of these 24 differentially expressed GMMs were linked to at least one differentially expressed SGB, indicating a strong association with obesity.

**Fig. 5:**
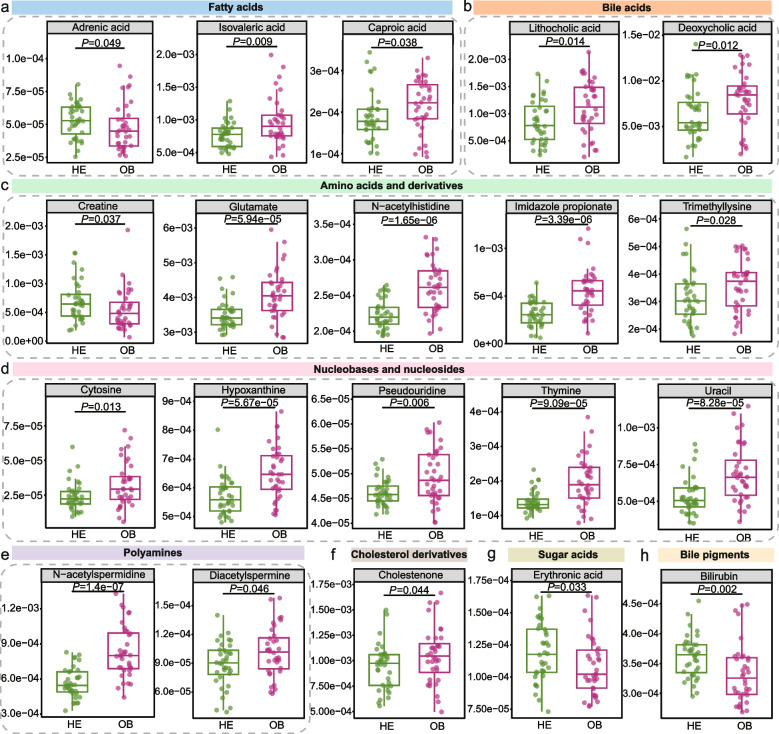
Changes in gut GPMs between healthy and obese adults. **a-h** The relative abundance of differential GPMs. The central line in the box plot denotes the median, while the box edges correspond to the interquartile range. The whiskers extend to values outside the upper and lower quartiles. Wilcoxon test was used to compare the differences between groups, and *P* < 0.05 was considered statistically significant

Computational prediction using MelonnPan suggested alterations in potential active gut metabolites in obese adults. A total of 20 differentially predicted metabolites were identified, including fatty acids, bile acids, amino acids and derivatives, nucleobases and nucleosides, polyamines, cholesterol derivatives, sugar acids, and bile pigments (Fig. 5a-h). Adrenic acid, creatine, and erythronic acid displayed significantly lower predicted relative abundances in the OB group (*P* < 0.05). In comparison, the remaining 18 metabolites, including bilirubin, caproic acid, cholestenone, cytosine, deoxycholic acid, diacetylspermine, glutamate, hypoxanthine, imidazole propionate, isovaleric acid, lithocholic acid, N-acetylhistidine, N-acetylspermidine, pseudouridine, thymine, trimethyllysine, and uracil, were predicted to be significantly elevated in the OB group (*P* < 0.05). These predicted compounds represent hypothesis-generating gut metabolic features associated with obesity that require further experimental validation.

### Receiver operating characteristic analysis (ROC) and correlation network of key differential SGBs, bacteriophages, GMMs, GPMs, and body composition indicators

**Fig. 6:**
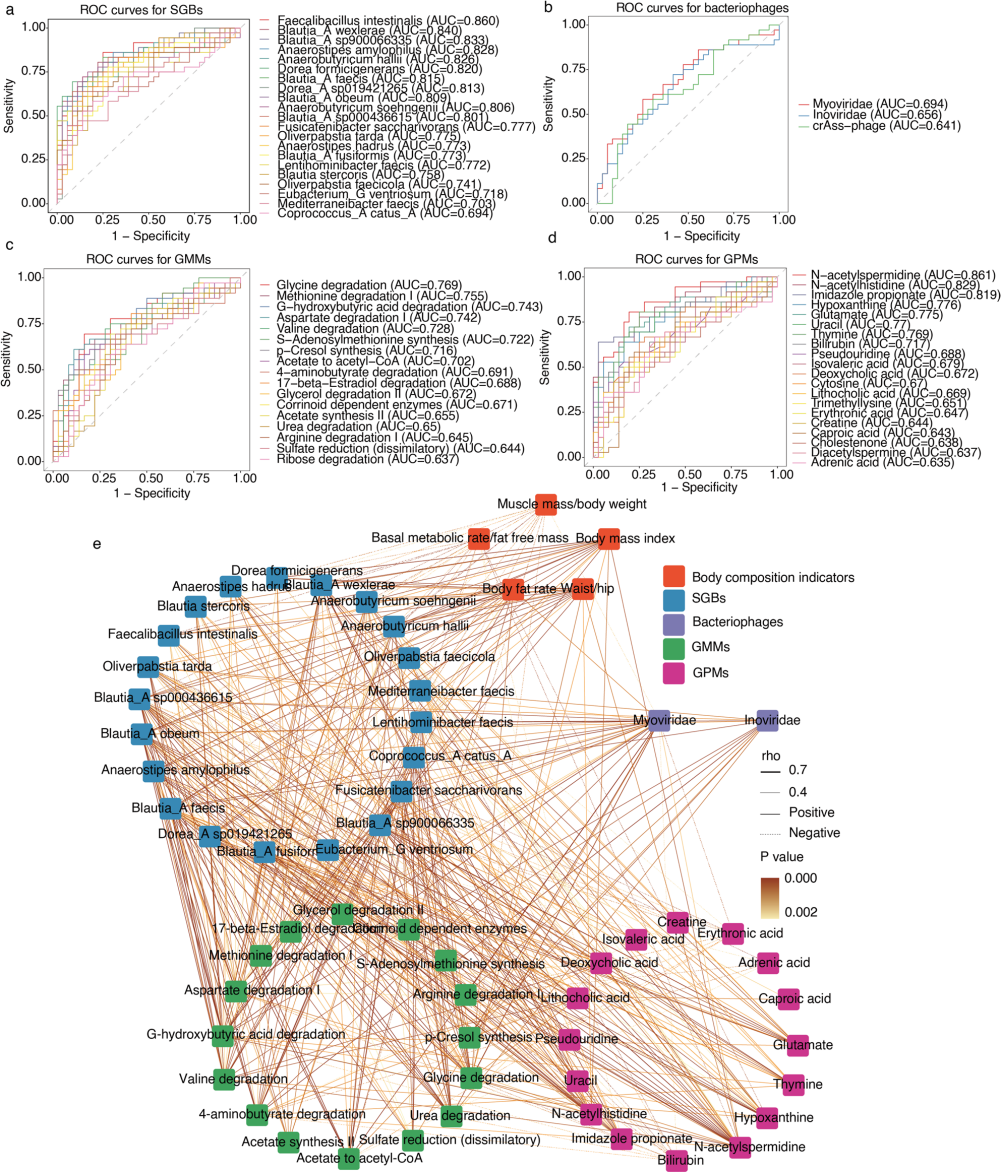
ROC curves of differential SGBs, bacteriophages, GMMs, GPMs, and correlation network of body composition indicators. **a-d** ROC curves of differential SGBs, bacteriophages, GMMs, and GPMs. The *x*- and *y*-axes represent 1 − specificity and sensitivity, respectively. Different colors indicate different features. **b** Spearman’s correlation coefficients between key differential SGBs, bacteriophages, GMMs, GPMs, and body composition indicators (including body mass index, body fat rate, waist-to-hip ratio, muscle mass-to-body weight ratio, and basal metabolic rate-to-fat-free mass ratio) (|*r*|≥ 0.4, *P* < 0.05). Blue, purple, green, pink, and orange squares represent SGBs, bacteriophages, GMMs, GPMs, and body composition indicators, respectively. Line thickness reflects the strength of the correlation; solid lines indicate positive correlations, and dashed lines indicate negative correlations. Color intensity corresponds to the *P*-value. Differences between features were assessed using the Wilcoxon test. *P* values were corrected using the Benjamini–Hochberg procedure, and *P* < 0.05 was considered statistically significant

To validate whether the key differential features identified in the previous study, including 21 SGBs, three bacteriophages, 17 GMMs, and 20 GPMs, could differentiate obese from healthy individuals, and to explore their multidimensional associations with body composition, the study performed ROC curve and correlation network analysis. The results demonstrated that most features possessed diagnostic potential for distinguishing obese from healthy adults. The area under the curve (AUC) values were as follows: SGBs, 0.694–0.860; bacteriophages, 0.641–0.694; GMMs, 0.637–0.769; and GPMs, 0.635–0.861. The 11 differential SGBs and three differential GPMs represented AUC values above 0.8, highlighting their strong discriminatory capacity for obesity. These included *Faecalibacillus intestinalis, Blautia wexlerae, Blautia* sp900066335*, Anaerostipes amylophilus, Anaerobutyricum hallii, Dorea formicigenerans, Blautia*_A *faecis, Dorea*_A sp019421265*, Blautia*_A *obeum, Anaerobutyricum soehngenii, Blautia*_A sp000436615*,* as well as N-acetylspermidine, N-acetylhistidine, and imidazole propionate (Fig. 6a–d).

A multi-omics correlation network was then constructed using Spearman's correlation to investigate the interrelationships among the features. After applying thresholds of *P* < 0.05 and |r|≥ 0.4, 21 SGBs, two bacteriophages, 16 GMMs, and 16 GPMs were retained (Fig. 6e, Table S3). Within the network, differentially expressed SGBs displayed the highest number of nodes, acting as central hubs linking differentially expressed bacteriophages, GMMs, GPMs, and body composition parameters. The six differential SGBs, *Dorea formicigenerans, Blautia*_A *wexlerae, Anaerobutyricum hallii, Blautia*_A sp900066335*, Dorea*_A sp019421265*,* and *Blautia*_A *obeum*, were significantly positively correlated with BMI (*R* = 0.50–0.56, *P* < 0.05), BFR (*R* = 0.43–0.56, *P* < 0.05), and WHR (*R* = 0.48–0.55, *P* < 0.05), while showing significant negative correlations with MM/BW (R = –0.55 to –0.41, *P* < 0.05) and BMR/FFM (*R* = –0.53 to –0.42, *P* < 0.05). All six SGBs also displayed significant positive associations with valine degradation (*R* = 0.41–0.57, *P* < 0.05) and N-acetylspermidine (*R* = 0.40–0.63, *P* < 0.05). Moreover, the latter three SGBs were consistently positively correlated with Myoviridae (*R* = 0.42–0.51, *P* < 0.05) and multiple metabolic modules, including aspartate degradation I, glycine degradation, urea degradation, 4-aminobutyrate degradation, G-hydroxybutyric acid degradation, and valine degradation (*R* = 0.41–0.59, *P* < 0.05). They also showed positive associations with several metabolites, including imidazole propionate, glutamate, uracil, hypoxanthine, and N-acetylspermidine (*R* = 0.42–0.63, *P* < 0.05). Regarding bacteriophages, both Inoviridae and Myoviridae showed significant positive correlations with p-Cresol synthesis, urea degradation, acetate-to-acetyl-CoA conversion, 4-aminobutyrate degradation, G-hydroxybutyric acid degradation, and arginine degradation I (*R* = 0.45–0.70, *P* < 0.05). The Myoviridae was positively correlated with BFR (*R* = 0.44, *P* = 6.61 × 10⁻^4^) and N-acetylspermidine (*R* = 0.41, *P* = 1.46 × 10⁻^3^), while showing negative correlations with MM/BW, erythronic acid, and bilirubin (*R* = –0.50 to –0.40, *P* < 0.05). At the level of GMMs, glycine degradation showed significant positive associations with BMI, BFR, and WHR (*R* = 0.43–0.46, *P* < 0.05), and was negatively correlated with MM/BW (*R* = –0.40, *P* = 6.67 × 10⁻^4^). Aspartate degradation I, methionine degradation I, and acetate to acetyl-CoA were positively associated with BFR (*R* = 0.41–0.48, *P* < 0.05), and the latter two were also negatively associated with MM/BW (*R* = –0.50, *P* < 0.05). Furthermore, Aspartate degradation I, glycine degradation, 4-aminobutyrate degradation, G-hydroxybutyric acid degradation, valine degradation, and arginine degradation I were all positively associated with N-acetylspermidine (*R* = 0.42–0.53, *P* < 0.05). Among them, the first four GMMs together with urea degradation were negatively associated with bilirubin (*R* = – 0.42 to – 0.46, *P* < 0.05), whereas all five modules except G-hydroxybutyric acid degradation were positively associated with lithocholic acid (*R* = 0.40–0.48, *P* < 0.05). 4-aminobutyrate degradation was positively associated with thymine (*R* = 0.43, *P* = 8.69 × 10⁻^4^) but inversely associated with creatine (*R* = – 0.40, *P* = 1.82 × 10⁻^3^), and arginine degradation I also observed a significant negative correlation with creatine (*R* = –0.40, *P* = 1.82 × 10⁻^3^). G-hydroxybutyric acid degradation was positively associated with thymine (*R* = 0.43, *P* = 9.66 × 10⁻^4^) and hypoxanthine (*R* = 0.43, *P* = 1.05 × 10⁻^3^), and valine degradation was also positively associated with hypoxanthine (*R* = 0.44, *P* = 8.33 × 10⁻^4^). At the level of GPMs, N-acetylhistidine and N-acetylspermidine were significantly positively correlated with BMI and WHR (*R* = 0.42–0.48, *P* < 0.05), whereas imidazole propionate, uracil, hypoxanthine, and N-acetylspermidine were significantly negatively associated with BMR/FFM (*R* = – 0.40 to – 0.50, *P* < 0.05).

Overall, the multi-omics correlation network illustrates coordinated interactions among SGBs, bacteriophages, GMMs, GPMs, and body composition indicators. These results indicate that the identified key features may contribute to obesity via multi-layered interactions and functional interdependencies.

## Discussion

In response to the escalating global obesity epidemic and its associated public health burden, modulation of the gut microbiota is increasingly recognized as a critical strategy for regulating host metabolic homeostasis and mitigating obesity-related phenotypes. Obesity is characterized by increased individual variability and high complexity, underscoring the need to identify intervention targets through multidimensional approaches. Most existing studies remain confined to single dimensions and lack systematic integration of the diverse features of the gut microbiome, limiting comprehensive understanding of the complex pathophysiological mechanisms of obesity and its potential therapeutic pathways. To address this gap, the present study integrated body composition, gut microbiota, bacteriophages, GMMs, and GPMs in obese adults. By constructing the first multidimensional integrated association network in this population, the study systematically characterized obesity-associated gut ecological features and identified potential features that require mechanistic investigation. The results showed that obese adults displayed not only characteristic alterations in body composition, including increased BMI, BFR, and WHR, alongside reduced MM/BW and BMR/FFM, but also significantly decreased gut microbial diversity. Distinct microbial communities, bacteriophages, and metabolic networks were found to undergo significant restructuring, with strong correlations to multiple body composition parameters. These findings deepen our understanding of the complex pathophysiological processes underlying obesity and provide the scientific basis for further development of personalized nutritional strategies, targeted microbiome modulation, and preventive interventions.

The gut microbiota has emerged as a critical determinant in the development and progression of obesity. Through modulation of microbial composition and metabolic activity, it influences host energy metabolism and body composition regulation. In this study, α-diversity of the gut microbiota was significantly reduced in the OB group, while β-diversity analyses revealed significant restructuring of the overall community. These findings align with previous observations of low-diversity microbial profiles associated with obesity [6–9]. Furthermore, the Bacillota/Bacteroidota ratio was elevated in obese adults, a shift consistently linked to enhanced energy harvest and fat accumulation [11, 13]. At the species level, 26 differential SGBs were identified, with 21 significantly enriched in obese adults, all of which belonged to the Bacillota, consistent with the increased Bacillota/Bacteroidota ratio. These SGBs showed positive correlations with BMI, BFR, and WHR, and negative correlations with MM/BW and BMR/FFM. Most enriched SGBs comprised closely related taxa within *Dorea* and *Blautia*, implicating these lineages in obesity-associated metabolic reprogramming. In particular, *Dorea formicigenerans* and *Dorea longicatena* were significantly increased in the OB group and positively correlated with BW and BMI, consistent with previous studies and suggesting their potential as biomarkers [42, 43]. Analyses of *Blautia* species revealed interspecific heterogeneity. Positive correlations between *Blautia obeum* and BW, BMI, waist, and hip circumference have been reported previously, in line with the findings of the present study [44]. In constrast, *Blautia wexlerae*, which is typically reduced in abundance [45, 46], was significantly elevated in our study. This discrepancy may reflect population-specific dietary patterns, sample characteristics, or strain-level functional diversity influencing metabolic activity [47]. Moreover, evidence regarding *Blautia fusiformis*, *Blautia stercoris*, and *Blautia faecis* remains limited, and the present findings provide a reference for future investigations. Additionally, Several SCFA-producing species were significantly enriched in the OB group, including *Anaerostipes hadrus*, *Anaerostipes amylophilus*, *Anaerobutyricum hallii*, and *Anaerobutyricum soehngenii*. Although this pattern contrasts with the previous reports [48–52], enhanced microbial energy harvest under caloric surplus may promote excess energy storage [53–55]. *Eubacterium ventriosum* and *Coprococcus catus* were enriched in the OB group and showed positive correlations with BMI, consistent with previous studies [56, 57]. In addition, several poorly characterized species were significantly enriched in the OB group (*Faecalibacillus intestinalis, Fusicatenibacter saccharivorans, Mediterraneibacter faecis, Oliverpabstia tarda,* and *Lentihominibacter faecis*), whereas others were significantly reduced (*Limisoma* sp000437795 *and Faecousia* sp003525905). These species may represent previously underrecognized features of obesity-associated microbial remodeling. Overall, this study offers an SGB-level framework linking gut microbiota alterations in obese adults to testable hypotheses and potential microbiome-based intervention targets.

Bacteriophages play a pivotal role in reshaping the gut microbiota in obesity by modulating bacterial host structure and function, thereby influencing host metabolism. In this study, α-diversity was significantly reduced in the OB group, with the overall bacteriophages community structure distinctly separated from that of healthy adults. The Shannon index of bacteriophages showed a strong positive correlation with that of bacterial SGBs, and Procrustes analysis revealed high concordance between their community structures. Consistent with recent reports [58–61], these observations indicate coordinated alterations in bacterial and bacteriophage communities in obesity, while causal relationships remain to be validated. Disruption of the bacterial community may reduce host resources or alter ecological niches, with downstream effects on phage abundance and diversity, while bacteriophages may influence bacterial community structure via host lysis, horizontal gene transfer, and microbial regulation [19–21]. Based on computational phage-host prediction analyses, key phage families previously linked to metabolic disorders (Siphoviridae, Myoviridae, crAss-phage, and Inoviridae) were predominantly associated with bacterial hosts within the Bacillota and Bacteroidota [20]. Moreover, among the 24 bacterial SGBs significantly enriched in the OB group, most were classified within Bacillota and concentrated in Lachnospiraceae, the primary predicted host family of these phages. Several studies have reported enrichment of Lachnospiraceae in obese individuals, accompanied by concurrent changes in the diversity and abundance of their associated phages [62–65]. These findings indicate parallel phage-bacterial alterations in obesity, although the inferred associations are computational and require experimental validation. Detailed composition difference revealed the shifts of bacteriophageome in obesity. The OB group exhibited increased relative abundance of Myoviridae and Inoviridae, potentially driven by xpanded bacterial host populations, which may facilitate phage infection and community restructuring. Similar increases in Myoviridae have been reported in children with obesity or metabolic syndrome, as well as in obese mouse models [64, 66], whereas reports on Inoviridae remain limited, rendering this observation potentially novel. In contrast, crAss-phage was significantly reduced in the OB group, consistent with findings in pediatric obesity and metabolic syndrome cohorts [67]. This reduction coincided with a decrease in *Limisoma* sp000437795 (Muribaculaceae), a potential host of crAss-phage, aligning with reported declines of Muribaculaceae under high-fat diets [68]. These results suggest coordinated phage-bacterial alteration characterized by parallel declines of crAss-phage and Muribaculaceae in obesity. These findings reinforce the regulatory role of phages in obesity-associated gut microbiota remodeling and highlight phage-bacterial variation as a potential factor in gut ecosystem stability and metabolic function, providing a theoretical basis for phage-based microbiome interventions.

Regarding predicted functions and metabolites, GMMs encoded by differential SGBs closely mirrored shifts in GPMs, collectively indicating a metabolic profile marked by increased energy harvesting, a pro-inflammatory state, and dysregulated signal transduction. In obese adults, enrichment modules involved in propionate production III, acetate synthesis II, and acetate to acetyl-CoA conversion suggested increased SCFA metabolic activity. At the same time, elevated degradation of branched-chain amino acids (BCAAs) (valine and isoleucine), accompanied by accumulation of BCAAs (isovaleric acid, caproic acid), indicated enhanced carbon–nitrogen flux redistribution via cross-metabolism, potentially contributing to increased intestinal energy availability. Consistent with previous reports, excessive SCFA production and elevated fecal BCAA levels have been linked to obesity-related metabolic disturbances and fat accumulation [53–55, 69]. A significant reduction in adrenic acid was observed, implicating obesity-associated lipid metabolic reprogramming along the gut-liver axis, although the reported change across tissues remains inconsistent and requires further validation [70–73]. Moreover, pathways such as aspartate, proline, methionine, glycine, and 4-aminobutyrate degradation were upregulated in obese adults, potentially contributing to increased glutamate production, together with enrichment of p-Cresol synthesis, a metabolite linked to impaired metabolic homeostasis [74]. Elevated S-adenosylmethionine synthesis and accumulation of polyamine-related metabolites (trimethyllysine, N-acetylspermidine, and diacetylspermine) may promote gut barrier dysfunction, inflammation, and altered transduction [75–77]. Predicted increases in imidazole propionate and N-acetylhistidine, together with reduced creatine levels, pointed to compromised intestinal energy buffering and barrier integrity, consistent with heightened inflammatory susceptibility [78–80]. Pathways related to bile acid transformation, including corrinoid-dependent enzymes, sulfate reduction, and 17-beta-estradiol degradation, were enriched in obese adults, accompanied by increased levels of lithocholic acid and deoxycholic acid. Elevated secondary bile acids can disrupt microbial membranes, promote lipopolysaccharide release, increase intestinal permeability, and trigger Toll-like receptors-mediated endotoxemia and inflammatory cytokines production [81]. Intestinal bilirubin levels were also decreased, potentially reflecting impaired bilirubin production driven by obesity-related alterations in gut microbiota composition and function, and may weaken host defense against oxidative stress and inflammation, exacerbating metabolic disturbances [82]. Furthermore, the accumulation of uracil, thymine, cytosine, pseudouridine, and hypoxanthine suggested restricted nucleotide metabolic flux, implying impaired functional coupling between the gut microbiota and host nucleotide metabolism [83, 84]. Moreover, reduced DOPAC synthesis and increased G-hydroxybutyric acid degradation may reflect impaired gut–brain axis signaling and altered microbial handling of neuroactive substrates in obesity [85, 86]. However, the GPMs presented in this study are computationally predicted metabolite profiles inferred from metagenomic data rather than experimentally measured metabolites. Accordingly, these findings should be regarded as hypothesis-generating signals, stable isotope tracing and targeted metabolomics are essential to validate these computational predictions and establish causal mechanisms.

Building on these findings, key differential SGBs, bacteriophages, GMMs, and GPMs were integrated to assess their diagnostic potential in distinguishing obese from healthy adults and their associations with body composition traits. Several SGBs (*Faecalibacillus intestinalis, Blautia wexlerae, Anaerostipes amylophilus, Dorea formicigenerans*) and GPMs (N-acetylspermidine, N-acetylhistidine, and imidazole propionate) exhibited strong discriminative performance (AUC > 0.8), indicating their potential as obesity-related biomarkers. Spearman correlation network analysis revealed extensive interconnections among differential features of the connecting nodes centered around SGBs. In particular, *Dorea formicigenerans*, *Blautia wexlerae*, and *Anaerobutyricum hallii* were positively correlated with BMI, BFR, and WHR and negatively correlated with MM/BW and BMR/FFM, suggesting their potential role as central hubs in obesity-associated energy metabolism and fat distribution [42, 43, 47, 87]. These SGBs were also positively associated with valine degradation and N-acetylspermidine, supporting microbial regulation of host metabolism via amino acid metabolism. Furthermore, *Blautia*_A sp900066335, *Dorea*_A sp019421265, and *Blautia*_A *obeum* were linked to *Myoviridae*, multiple amino acid degradation pathways (e.g., aspartate degradation I, glycine degradation, urea degradation, 4-aminobutyrate degradation, G-hydroxybutyric acid degradation, valine degradation), and metabolites (e.g., imidazole propionate, glutamate, uracil, hypoxanthine, N-acetylspermidine), highlighting the central role of *Dorea* and *Blautia* in host metabolic regulation. At the phage level, Inoviridae and Myoviridae were significantly associated with multiple amino acid degradation pathways, including p-Cresol synthesis, urea degradation, acetate to acetyl-CoA conversion, 4-aminobutyrate degradation, G-hydroxybutyric acid degradation, and arginine degradation I. Notably, Myoviridae displayed positive correlations with BFR and N-acetylspermidine and negative correlations with MM/BW, erythronic acid, and bilirubin, suggesting an indirect contribution of phages to obesity phenotypes via microbial metabolic modulation [66, 67]. GMMs analysis showed that glycine degradation, aspartate degradation, I, 4-aminobutyrate degradation, and G-hydroxybutyric acid degradation were positively correlated with BFR, N-acetylspermidine, and bilirubin, indicating indirect contribution to obesity-related metabolic phenotypes via amino acid and small molecule metabolism [88–91]. Although bilirubin has been reported to inversely correlate with BMI and FM [92], this association did not meet our screening criteria. Additionally, negative correlations between 4-aminobutyrate and arginine degradation and creatine suggest microbial modulation of muscle energy homeostasis. At the GPMs level, N-acetylhistidine and N-acetylspermidine were positively correlated with BMI and WHR, whereas imidazole propionate and hypoxanthine were negatively correlated with BMR/FFM. Imidazole propionate has been implicated in insulin resistance via disruption of insulin signaling [79]. Hypoxanthine, reflecting altered purine metabolism, demonstrated a positive association with BMI and may represent an adverse metabolic biomarker [93]. Although the mechanisms linking N-acetylhistidine and N-acetylspermine to obesity remain unclear, these amino acid and polyamine alterations likely reflect microbiota-driven metabolic network dysregulation.

This study provides a foundation for microbiome-based biomarkers discovery and therapeutic target exploration in obese adults, but several limitations warrant consideration. The limited sample size and population composition restrict generalizability and statistical power, potentially reducing sensitivity to weaker associations in high-dimensional multi-omics analysis. Given geographic, dietary, and lifestyle heterogeneity, these findings should be interpreted as exploratory and require validation in larger, multicenter, and longitudinal cohorts to support causal or mechanistic inference. Moreover, bacteriophage profiling is further constrained by incomplete database, limited host prediction accuracy, which may underestimate phage diversity and function. In addition, GPMs represent computational predictions and require validation through targeted metabolomics before biological interpretation or clinical translation. Despite these limitations, the integrative framework presented here establishes a theoretical basis for obesity prevention and intervention via gut microbiota modulation. Future studies should combine large-scale validation with multi-omics integration, machine learning, and network modeling, alongside targeted verification of candidate genes, pathways, and metabolites, and incorporation of host genetics and diet, to enable precise microbiome-based intervention strategies.

## Conclusions

This study characterized multi-level associations between gut microbiome features and body composition in obese adults through multi-omics integration. Comprehensive analysis identified 21 key differential SGBs, 2 bacteriophages, 16 GMMs, and 16 GPMs, and showed distinct associations with body composition indicators. These findings offer hypothesis-generating insights into gut microbiome- and metabolism-related features in obesity. Critically, the cross-sectional design and reliance on computational predictions necessitate prospective validation and experimental mechanistic studies before clinical translation.

## Supplementary Information

Supplementary Material 1.

Supplementary Material 2.

Supplementary Material 3.

## Data Availability

All data generated or analysed during this study are included in this published article and its supplementary information files. The sequence data set has been deposited in the China National GeneBank DataBase under the accession number CNP0008229 (https://db.cngb.org/data_resources/project?query=CNP0008229).
