# Supplementary material for: Integrative profiling of gut microbiome, bacteriophagenome, and predicted metabolome in obese adults: novel insights into intervention targets
Source: BMC Microbiol. 2026 Jan 29;26:226. doi: 10.1186/s12866-025-04682-1 (PMC12973561; doi:10.1186/s12866-025-04682-1)
Supplement: Supplementary file 1 — Supplementary Material 1. [file 12866_2025_4682_MOESM1_ESM.docx]

**Table S1. P value, fold change, and VIP of 26 differential SGBs.**

| **Species-level gut microbiota** | ***P* value** | **FC (HE/OB)** | **VIP** |
| --- | --- | --- | --- |
| *Faecalibacillus intestinalis* | 1.58E-07 | 0.24 | 2.84 |
| *Blautia_*A *wexlerae* | 7.43E-07 | 0.30 | 2.52 |
| *Dorea formicigenerans* | 3.04E-06 | 0.23 | 2.47 |
| *Blautia_*A *obeum* | 6.82E-06 | 0.31 | 2.40 |
| *Anaerobutyricum hallii* | 1.95E-06 | 0.21 | 2.34 |
| *Dorea_*A *longicatena* | 2.81E-04 | 0.24 | 2.29 |
| *Blautia_*A *fusiformis* | 6.86E-05 | 0.30 | 2.24 |
| *Dorea_*A *sp019421265* | 4.95E-06 | 0.46 | 2.21 |
| *Fusicatenibacter saccharivorans* | 5.40E-05 | 0.43 | 2.20 |
| *Blautia_*A *faecis* | 4.45E-06 | 0.39 | 1.96 |
| *Anaerostipes hadrus* | 6.86E-05 | 0.33 | 1.94 |
| *Anaerobutyricum soehngenii* | 8.42E-06 | 0.26 | 1.90 |
| *Coprococcus_*A *catus_*A | 4.62E-03 | 0.46 | 1.86 |
| *Eubacterium_*G *sp000435815* | 4.51E-04 | 0.22 | 1.84 |
| *Blautia stercoris* | 1.65E-04 | 0.26 | 1.82 |
| *Oliverpabstia faecicola* | 4.51E-04 | 0.26 | 1.81 |
| *Streptococcus salivarius* | 4.30E-03 | 0.30 | 1.81 |
| *Blautia_*A *sp000436615* | 1.15E-05 | 0.21 | 1.79 |
| *Mediterraneibacter faecis* | 3.11E-03 | 0.39 | 1.75 |
| *Oliverpabstia tarda* | 6.23E-05 | 0.29 | 1.70 |
| *Lentihominibacter faecis* | 7.19E-05 | 0.18 | 1.68 |
| *Anaerostipes amylophilus* | 1.75E-06 | 0.12 | 1.67 |
| *Blautia_*A *sp900066335* | 1.24E-06 | 0.21 | 1.67 |
| *Eubacterium_*G *ventriosum* | 1.46E-03 | 0.34 | 1.66 |
| *Limisoma sp000437795* | 6.54E-05 | 3.72 | 1.52 |
| *Faecousia sp003525905* | 3.47E-02 | 4.52 | 1.52 |
