# Supplementary material for: Integrative profiling of gut microbiome, bacteriophagenome, and predicted metabolome in obese adults: novel insights into intervention targets
Source: BMC Microbiol. 2026 Jan 29;26:226. doi: 10.1186/s12866-025-04682-1 (PMC12973561; doi:10.1186/s12866-025-04682-1)
Supplement: Supplementary file 2 — Supplementary Material 2. [file 12866_2025_4682_MOESM2_ESM.docx]

**Table S2. P value of 24 differential GMMs.**

| **GMMs** | ***P* value** | **Change in OB group** |
| --- | --- | --- |
| p-Cresol synthesis | 1.64E-03 | Up-regulated |
| Tyrosine degradation II | 3.28E-02 |  |
| Aspartate degradation I | 4.14E-04 |  |
| Methionine degradation I | 2.07E-04 |  |
| Isoleucine degradation | 3.60E-03 |  |
| Glycine degradation | 9.09E-05 |  |
| Urea degradation | 2.93E-02 |  |
| Propionate production III | 2.93E-02 |  |
| Trehalose degradation | 3.10E-02 |  |
| Glycerol degradation II | 1.22E-02 |  |
| Corrinoid dependent enzymes | 1.30E-02 |  |
| Sulfate reduction (dissimilatory) | 3.67E-02 |  |
| Acetate to acetyl-CoA | 3.23E-03 |  |
| Acetate synthesis II | 2.39E-02 |  |
| 4-aminobutyrate degradation | 5.50E-03 |  |
| Valine degradation | 8.75E-04 |  |
| Inositol synthesis | 1.52E-02 |  |
| G-hydroxybutyric acid degradation | 3.97E-04 |  |
| Proline degradation | 2.06E-02 |  |
| S-Adenosylmethionine synthesis | 1.20E-03 |  |
| 17-beta-Estradiol degradation | 6.31E-03 |  |
| Arginine degradation I | 3.47E-02 |  |
| Ribose degradation | 4.56E-02 | Down-regulated |
| DOPAC synthesis | 3.88E-02 |  |
