# Supplementary material for: Integrative profiling of gut microbiome, bacteriophagenome, and predicted metabolome in obese adults: novel insights into intervention targets
Source: BMC Microbiol. 2026 Jan 29;26:226. doi: 10.1186/s12866-025-04682-1 (PMC12973561; doi:10.1186/s12866-025-04682-1)
Supplement: Supplementary file 3 — Supplementary Material 3. [file 12866_2025_4682_MOESM3_ESM.docx]

**Table S3. P value and Spearman correlation coefficient between SGBs, bacteriophages, GMMs, GPMs, and body composition indicators.**

| **Node 1** | **Node 2** | ***P* value** | **correlation coefficient** |
| --- | --- | --- | --- |
| *Lentihominibacter faecis* | Inoviridae | 2.30E-05 | 0.56 |
| *Lentihominibacter faecis* | Myoviridae | 1.71E-05 | 0.57 |
| *Coprococcus_*A *catus_*A | Myoviridae | 1.05E-04 | 0.51 |
| *Fusicatenibacter saccharivorans* | Myoviridae | 1.87E-04 | 0.49 |
| *Blautia_*A *sp900066335* | Myoviridae | 9.07E-05 | 0.51 |
| *Eubacterium_G ventriosum* | Myoviridae | 8.15E-04 | 0.44 |
| *Blautia_*A *fusiformis* | Myoviridae | 4.27E-04 | 0.46 |
| *Dorea_*A *sp019421265* | Myoviridae | 1.26E-03 | 0.42 |
| *Blautia_*A *faecis* | Inoviridae | 6.01E-05 | 0.53 |
| *Blautia_*A *faecis* | Myoviridae | 6.25E-06 | 0.59 |
| *Anaerostipes amylophilus* | Myoviridae | 6.97E-04 | 0.44 |
| *Blautia_*A *obeum* | Inoviridae | 1.02E-03 | 0.43 |
| *Blautia_*A *obeum* | Myoviridae | 2.56E-04 | 0.48 |
| *Blautia_*A *sp000436615* | Inoviridae | 8.25E-04 | 0.44 |
| *Blautia_*A *sp000436615* | Myoviridae | 6.65E-05 | 0.52 |
| *Oliverpabstia tarda* | Inoviridae | 5.82E-04 | 0.45 |
| *Oliverpabstia tarda* | Myoviridae | 1.04E-03 | 0.43 |
| *Lentihominibacter faecis* | p-Cresol synthesis | 9.68E-04 | 0.43 |
| *Lentihominibacter faecis* | Glycine degradation | 1.15E-04 | 0.51 |
| *Lentihominibacter faecis* | Urea degradation | 2.87E-05 | 0.55 |
| *Lentihominibacter faecis* | Sulfate reduction (dissimilatory) | 1.25E-04 | 0.5 |
| *Lentihominibacter faecis* | Acetate to acetyl-CoA | 4.07E-05 | 0.54 |
| *Lentihominibacter faecis* | Acetate synthesis II | 9.68E-04 | 0.43 |
| *Lentihominibacter faecis* | 4-aminobutyrate degradation | 5.49E-06 | 0.6 |
| *Lentihominibacter faecis* | Valine degradation | 2.58E-04 | 0.48 |
| *Lentihominibacter faecis* | G-hydroxybutyric acid degradation | 2.14E-05 | 0.56 |
| *Coprococcus_*A *catus_*A | Aspartate degradation I | 1.64E-03 | 0.41 |
| *Coprococcus_*A *catus*_A | Glycine degradation | 1.25E-04 | 0.5 |
| *Coprococcus*_A *catus*_A | Urea degradation | 1.90E-04 | 0.49 |
| *Coprococcus*_A *catus*_A | Sulfate reduction (dissimilatory) | 1.73E-05 | 0.56 |
| *Coprococcus*_A *catus*_A | Acetate to acetyl-CoA | 1.34E-04 | 0.5 |
| *Coprococcus*_A *catus*_A | Acetate synthesis II | 4.41E-04 | 0.46 |
| *Coprococcus*_A *catus*_A | 4-aminobutyrate degradation | 1.25E-04 | 0.5 |
| *Coprococcus*_A *catus*_A | Valine degradation | 1.08E-04 | 0.51 |
| *Coprococcus*_A *catus*_A | G-hydroxybutyric acid degradation | 4.52E-05 | 0.54 |
| *Faecalibacillus intestinalis* | Aspartate degradation I | 1.71E-03 | 0.41 |
| *Faecalibacillus intestinalis* | Glycine degradation | 1.59E-03 | 0.41 |
| *Faecalibacillus intestinalis* | Sulfate reduction (dissimilatory) | 1.79E-03 | 0.4 |
| *Faecalibacillus intestinalis* | Valine degradation | 1.59E-03 | 0.41 |
| *Faecalibacillus intestinalis* | G-hydroxybutyric acid degradation | 6.75E-04 | 0.44 |
| *Blautia stercoris* | Aspartate degradation I | 7.81E-04 | 0.44 |
| *Blautia stercoris* | Glycine degradation | 7.51E-04 | 0.44 |
| *Blautia stercoris* | Urea degradation | 8.06E-04 | 0.44 |
| *Blautia stercoris* | Sulfate reduction (dissimilatory) | 1.99E-03 | 0.4 |
| *Blautia stercoris* | Acetate synthesis II | 7.65E-04 | 0.44 |
| *Blautia stercoris* | Valine degradation | 3.38E-04 | 0.47 |
| *Blautia stercoris* | G-hydroxybutyric acid degradation | 3.55E-04 | 0.47 |
| *Anaerostipes hadrus* | Aspartate degradation I | 1.71E-03 | 0.41 |
| *Anaerostipes hadrus* | Methionine degradation I | 7.97E-04 | 0.44 |
| *Anaerostipes hadrus* | Glycine degradation | 9.95E-04 | 0.43 |
| *Anaerostipes hadrus* | Urea degradation | 3.57E-04 | 0.47 |
| *Anaerostipes hadrus* | Valine degradation | 1.69E-03 | 0.41 |
| *Anaerostipes hadrus* | G-hydroxybutyric acid degradation | 9.33E-04 | 0.43 |
| *Dorea formicigenerans* | Acetate synthesis II | 5.90E-04 | 0.45 |
| *Dorea formicigenerans* | Valine degradation | 1.65E-03 | 0.41 |
| *Dorea formicigenerans* | 17-beta-Estradiol degradation | 1.27E-03 | 0.42 |
| *Fusicatenibacter saccharivorans* | p-Cresol synthesis | 3.34E-04 | 0.47 |
| *Fusicatenibacter saccharivorans* | Aspartate degradation I | 6.77E-07 | 0.63 |
| *Fusicatenibacter saccharivorans* | Methionine degradation I | 1.96E-04 | 0.49 |
| *Fusicatenibacter saccharivorans* | Glycine degradation | 4.70E-05 | 0.54 |
| *Fusicatenibacter saccharivorans* | Urea degradation | 2.72E-05 | 0.55 |
| *Fusicatenibacter saccharivorans* | Glycerol degradation II | 1.34E-04 | 0.5 |
| *Fusicatenibacter saccharivorans* | Corrinoid dependent enzymes | 5.69E-04 | 0.45 |
| *Fusicatenibacter saccharivorans* | Sulfate reduction (dissimilatory) | 3.28E-04 | 0.47 |
| *Fusicatenibacter saccharivorans* | Acetate to acetyl-CoA | 3.48E-05 | 0.55 |
| *Fusicatenibacter saccharivorans* | 4-aminobutyrate degradation | 1.98E-04 | 0.49 |
| *Fusicatenibacter saccharivorans* | Valine degradation | 7.82E-05 | 0.52 |
| *Fusicatenibacter saccharivorans* | G-hydroxybutyric acid degradation | 1.51E-05 | 0.57 |
| *Blautia*_A *sp900066335* | p-Cresol synthesis | 1.75E-03 | 0.41 |
| *Blautia*_A *sp900066335* | Aspartate degradation I | 4.26E-04 | 0.46 |
| *Blautia*_A *sp900066335* | Methionine degradation I | 1.70E-03 | 0.41 |
| *Blautia*_A *sp900066335* | Glycine degradation | 2.37E-04 | 0.48 |
| *Blautia*_A *sp900066335* | Urea degradation | 2.87E-05 | 0.55 |
| *Blautia*_A *sp900066335* | Acetate to acetyl-CoA | 3.47E-04 | 0.47 |
| *Blautia*_A *sp900066335* | 4-aminobutyrate degradation | 4.21E-05 | 0.54 |
| *Blautia*_A *sp900066335* | Valine degradation | 1.89E-04 | 0.49 |
| *Blautia*_A *sp900066335* | G-hydroxybutyric acid degradation | 5.04E-05 | 0.53 |
| *Blautia*_A *wexlerae* | Aspartate degradation I | 6.03E-04 | 0.45 |
| *Blautia*_A *wexlerae* | Methionine degradation I | 1.75E-04 | 0.49 |
| *Blautia*_A *wexlerae* | Glycine degradation | 1.96E-04 | 0.49 |
| *Blautia*_A *wexlerae* | Urea degradation | 1.19E-03 | 0.42 |
| *Blautia*_A *wexlerae* | Acetate to acetyl-CoA | 1.12E-03 | 0.42 |
| *Blautia*_A *wexlerae* | 4-aminobutyrate degradation | 1.60E-04 | 0.49 |
| *Blautia*_A *wexlerae* | Valine degradation | 1.25E-04 | 0.5 |
| *Blautia*_A *wexlerae* | G-hydroxybutyric acid degradation | 5.77E-04 | 0.45 |
| *Eubacterium_G ventriosum* | Glycine degradation | 9.66E-04 | 0.43 |
| *Eubacterium_G ventriosum* | G-hydroxybutyric acid degradation | 1.20E-03 | 0.42 |
| *Anaerobutyricum soehngenii* | Aspartate degradation I | 6.92E-04 | 0.44 |
| *Anaerobutyricum soehngenii* | Methionine degradation I | 1.56E-03 | 0.41 |
| *Anaerobutyricum soehngenii* | Glycine degradation | 1.11E-03 | 0.42 |
| *Anaerobutyricum soehngenii* | Urea degradation | 3.04E-04 | 0.47 |
| *Anaerobutyricum soehngenii* | Sulfate reduction (dissimilatory) | 1.79E-03 | 0.4 |
| *Anaerobutyricum soehngenii* | Acetate to acetyl-CoA | 9.84E-04 | 0.43 |
| *Anaerobutyricum soehngenii* | Acetate synthesis II | 4.63E-04 | 0.46 |
| *Anaerobutyricum soehngenii* | 4-aminobutyrate degradation | 1.96E-04 | 0.49 |
| *Anaerobutyricum soehngenii* | Valine degradation | 2.20E-04 | 0.48 |
| *Anaerobutyricum soehngenii* | G-hydroxybutyric acid degradation | 3.00E-04 | 0.47 |
| *Blautia*_A *fusiformis* | Aspartate degradation I | 3.55E-04 | 0.47 |
| *Blautia*_A *fusiformis* | Urea degradation | 6.36E-06 | 0.59 |
| *Blautia*_A *fusiformis* | 4-aminobutyrate degradation | 1.41E-03 | 0.41 |
| *Blautia*_A *fusiformis* | Valine degradation | 6.96E-04 | 0.44 |
| *Blautia*_A *fusiformis* | G-hydroxybutyric acid degradation | 1.87E-04 | 0.49 |
| *Blautia*_A *fusiformis* | S-Adenosylmethionine synthesis | 6.03E-04 | 0.45 |
| *Anaerobutyricum hallii* | Aspartate degradation I | 1.53E-03 | 0.41 |
| *Anaerobutyricum hallii* | Methionine degradation I | 3.66E-04 | 0.47 |
| *Anaerobutyricum hallii* | Glycine degradation | 1.21E-04 | 0.51 |
| *Anaerobutyricum hallii* | Urea degradation | 4.19E-04 | 0.46 |
| *Anaerobutyricum hallii* | Acetate to acetyl-CoA | 1.80E-04 | 0.49 |
| *Anaerobutyricum hallii* | 4-aminobutyrate degradation | 2.56E-04 | 0.48 |
| *Anaerobutyricum hallii* | Valine degradation | 1.53E-04 | 0.5 |
| *Anaerobutyricum hallii* | G-hydroxybutyric acid degradation | 1.91E-03 | 0.4 |
| *Dorea*_A *sp019421265* | Aspartate degradation I | 1.89E-04 | 0.49 |
| *Dorea*_A *sp019421265* | Methionine degradation I | 1.53E-04 | 0.5 |
| *Dorea*_A *sp019421265* | Glycine degradation | 9.98E-04 | 0.43 |
| *Dorea*_A *sp019421265* | Urea degradation | 6.40E-04 | 0.45 |
| *Dorea*_A *sp019421265* | Glycerol degradation II | 6.67E-04 | 0.44 |
| *Dorea*_A *sp019421265* | Sulfate reduction (dissimilatory) | 1.26E-03 | 0.42 |
| *Dorea*_A *sp019421265* | 4-aminobutyrate degradation | 1.57E-03 | 0.41 |
| *Dorea*_A *sp019421265* | Valine degradation | 2.97E-04 | 0.47 |
| *Dorea*_A *sp019421265* | G-hydroxybutyric acid degradation | 5.40E-05 | 0.53 |
| *Blautia*_A *faecis* | p-Cresol synthesis | 3.55E-05 | 0.54 |
| *Blautia*_A *faecis* | Aspartate degradation I | 6.25E-06 | 0.59 |
| *Blautia*_A *faecis* | Methionine degradation I | 3.00E-04 | 0.47 |
| *Blautia*_A *faecis* | Glycine degradation | 0.00E+00 | 0.65 |
| *Blautia*_A *faecis* | Urea degradation | 1.76E-06 | 0.62 |
| *Blautia*_A *faecis* | Glycerol degradation II | 5.95E-05 | 0.53 |
| *Blautia*_A *faecis* | Sulfate reduction (dissimilatory) | 1.05E-04 | 0.51 |
| *Blautia*_A *faecis* | Acetate to acetyl-CoA | 2.64E-06 | 0.61 |
| *Blautia*_A *faecis* | 4-aminobutyrate degradation | 0.00E+00 | 0.65 |
| *Blautia*_A *faecis* | Valine degradation | 0.00E+00 | 0.67 |
| *Blautia*_A *faecis* | G-hydroxybutyric acid degradation | 0.00E+00 | 0.66 |
| *Blautia*_A *faecis* | Arginine degradation I | 9.66E-04 | 0.43 |
| *Anaerostipes amylophilus* | Aspartate degradation I | 1.23E-03 | 0.42 |
| *Anaerostipes amylophilus* | Glycine degradation | 1.34E-03 | 0.42 |
| *Anaerostipes amylophilus* | Urea degradation | 2.20E-04 | 0.48 |
| *Anaerostipes amylophilus* | 4-aminobutyrate degradation | 1.72E-03 | 0.41 |
| *Anaerostipes amylophilus* | Valine degradation | 6.92E-04 | 0.44 |
| *Anaerostipes amylophilus* | G-hydroxybutyric acid degradation | 5.04E-05 | 0.53 |
| *Blautia*_A *obeum* | p-Cresol synthesis | 7.51E-04 | 0.44 |
| *Blautia*_A *obeum* | Aspartate degradation I | 8.43E-04 | 0.43 |
| *Blautia*_A *obeum* | Glycine degradation | 1.09E-05 | 0.58 |
| *Blautia*_A *obeum* | Urea degradation | 6.79E-05 | 0.52 |
| *Blautia*_A *obeum* | Acetate to acetyl-CoA | 1.71E-05 | 0.57 |
| *Blautia*_A *obeum* | Acetate synthesis II | 1.28E-03 | 0.42 |
| *Blautia*_A *obeum* | 4-aminobutyrate degradation | 6.36E-06 | 0.59 |
| *Blautia*_A *obeum* | Valine degradation | 1.39E-05 | 0.57 |
| *Blautia*_A *obeum* | G-hydroxybutyric acid degradation | 3.43E-05 | 0.55 |
| *Blautia*_A *obeum* | Arginine degradation I | 1.37E-03 | 0.42 |
| *Blautia*_A *sp000436615* | p-Cresol synthesis | 6.51E-04 | 0.45 |
| *Blautia*_A *sp000436615* | Aspartate degradation I | 2.26E-04 | 0.48 |
| *Blautia*_A *sp000436615* | Methionine degradation I | 1.83E-03 | 0.4 |
| *Blautia*_A *sp000436615* | Glycine degradation | 2.54E-05 | 0.56 |
| *Blautia*_A *sp000436615* | Urea degradation | 7.49E-06 | 0.59 |
| *Blautia*_A *sp000436615* | Glycerol degradation II | 5.97E-04 | 0.45 |
| *Blautia*_A *sp000436615* | Sulfate reduction (dissimilatory) | 1.11E-03 | 0.42 |
| *Blautia*_A *sp000436615* | Acetate to acetyl-CoA | 4.51E-06 | 0.6 |
| *Blautia*_A *sp000436615* | Acetate synthesis II | 4.03E-04 | 0.46 |
| *Blautia*_A *sp000436615* | 4-aminobutyrate degradation | 2.41E-04 | 0.48 |
| *Blautia*_A *sp000436615* | Valine degradation | 8.92E-05 | 0.51 |
| *Blautia*_A *sp000436615* | G-hydroxybutyric acid degradation | 2.61E-05 | 0.56 |
| *Blautia*_A *sp000436615* | Arginine degradation I | 4.64E-05 | 0.54 |
| *Oliverpabstia faecicola* | 4-aminobutyrate degradation | 7.51E-04 | 0.44 |
| *Oliverpabstia tarda* | Glycine degradation | 1.52E-03 | 0.41 |
| *Oliverpabstia tarda* | Acetate synthesis II | 2.74E-04 | 0.47 |
| *Oliverpabstia tarda* | 4-aminobutyrate degradation | 3.58E-05 | 0.54 |
| *Oliverpabstia tarda* | Valine degradation | 1.96E-04 | 0.49 |
| *Oliverpabstia tarda* | G-hydroxybutyric acid degradation | 2.52E-04 | 0.48 |
| *Lentihominibacter faecis* | Glutamate | 6.60E-04 | 0.44 |
| *Lentihominibacter faecis* | Thymine | 1.33E-03 | 0.42 |
| *Lentihominibacter faecis* | Hypoxanthine | 1.99E-03 | 0.4 |
| *Lentihominibacter faecis* | N-acetylspermidine | 8.69E-04 | 0.43 |
| *Lentihominibacter faecis* | Bilirubin | 9.02E-04 | -0.43 |
| *Coprococcus*_A *catus*_A | N-acetylspermidine | 3.91E-04 | 0.46 |
| *Faecalibacillus intestinalis* | Imidazole propionate | 8.20E-04 | 0.44 |
| *Faecalibacillus intestinalis* | N-acetylhistidine | 3.41E-04 | 0.47 |
| *Faecalibacillus intestinalis* | Hypoxanthine | 1.24E-03 | 0.42 |
| *Faecalibacillus intestinalis* | N-acetylspermidine | 3.84E-05 | 0.54 |
| *Faecalibacillus intestinalis* | Bilirubin | 1.69E-03 | -0.41 |
| *Blautia stercoris* | Hypoxanthine | 1.24E-03 | 0.42 |
| *Blautia stercoris* | N-acetylspermidine | 9.33E-04 | 0.43 |
| *Anaerostipes hadrus* | Imidazole propionate | 5.95E-05 | 0.53 |
| *Anaerostipes hadrus* | Glutamate | 6.96E-04 | 0.44 |
| *Anaerostipes hadrus* | N-acetylhistidine | 1.08E-03 | 0.42 |
| *Anaerostipes hadrus* | Uracil | 5.38E-04 | 0.45 |
| *Anaerostipes hadrus* | Hypoxanthine | 1.50E-03 | 0.41 |
| *Anaerostipes hadrus* | Pseudouridine | 5.63E-04 | 0.45 |
| *Anaerostipes hadrus* | N-acetylspermidine | 5.20E-05 | 0.53 |
| *Dorea formicigenerans* | N-acetylspermidine | 1.58E-03 | 0.41 |
| *Mediterraneibacter faecis* | Hypoxanthine | 1.23E-03 | 0.42 |
| *Mediterraneibacter faecis* | N-acetylspermidine | 9.80E-04 | 0.43 |
| *Fusicatenibacter saccharivorans* | Lithocholic acid | 1.24E-03 | 0.42 |
| *Fusicatenibacter saccharivorans* | Deoxycholic acid | 1.56E-03 | 0.41 |
| *Fusicatenibacter saccharivorans* | Imidazole propionate | 5.97E-04 | 0.45 |
| *Fusicatenibacter saccharivorans* | N-acetylhistidine | 3.47E-04 | 0.47 |
| *Fusicatenibacter saccharivorans* | Hypoxanthine | 1.16E-03 | 0.42 |
| *Fusicatenibacter saccharivorans* | N-acetylspermidine | 5.97E-05 | 0.53 |
| *Blautia*_A *sp900066335* | Isovaleric acid | 7.99E-04 | 0.44 |
| *Blautia*_A *sp900066335* | Deoxycholic acid | 5.69E-04 | 0.45 |
| *Blautia*_A *sp900066335* | Imidazole propionate | 2.86E-04 | 0.47 |
| *Blautia*_A *sp900066335* | Glutamate | 1.13E-04 | 0.51 |
| *Blautia*_A *sp900066335* | Creatine | 5.77E-04 | -0.45 |
| *Blautia*_A *sp900066335* | N-acetylhistidine | 9.09E-05 | 0.51 |
| *Blautia*_A *sp900066335* | Uracil | 2.98E-04 | 0.47 |
| *Blautia*_A *sp900066335* | Thymine | 1.56E-04 | 0.5 |
| *Blautia*_A *sp900066335* | Hypoxanthine | 4.07E-05 | 0.54 |
| *Blautia*_A *sp900066335* | Pseudouridine | 2.91E-04 | 0.47 |
| *Blautia*_A *sp900066335* | N-acetylspermidine | 1.39E-05 | 0.57 |
| *Blautia*_A *sp900066335* | Erythronic acid | 4.17E-04 | -0.46 |
| *Blautia*_A *sp900066335* | Bilirubin | 6.17E-05 | -0.52 |
| *Blautia*_A *wexlerae* | Imidazole propionate | 7.45E-06 | 0.59 |
| *Blautia*_A *wexlerae* | Glutamate | 1.54E-04 | 0.5 |
| *Blautia*_A *wexlerae* | N-acetylhistidine | 5.04E-05 | 0.53 |
| *Blautia*_A *wexlerae* | Uracil | 5.95E-05 | 0.53 |
| *Blautia*_A *wexlerae* | Thymine | 1.12E-03 | 0.42 |
| *Blautia*_A *wexlerae* | Hypoxanthine | 2.87E-05 | 0.55 |
| *Blautia*_A *wexlerae* | N-acetylspermidine | 2.60E-06 | 0.61 |
| *Anaerobutyricum soehngenii* | Imidazole propionate | 1.34E-03 | 0.42 |
| *Anaerobutyricum soehngenii* | Glutamate | 7.99E-04 | 0.44 |
| *Anaerobutyricum soehngenii* | N-acetylhistidine | 1.45E-03 | 0.41 |
| *Anaerobutyricum soehngenii* | Hypoxanthine | 3.34E-04 | 0.47 |
| *Anaerobutyricum soehngenii* | N-acetylspermidine | 2.09E-04 | 0.48 |
| *Blautia*_A *fusiformis* | Deoxycholic acid | 8.47E-04 | 0.43 |
| *Blautia*_A *fusiformis* | Glutamate | 1.25E-04 | 0.5 |
| *Blautia*_A *fusiformis* | N-acetylhistidine | 2.50E-04 | 0.48 |
| *Blautia*_A *fusiformis* | Uracil | 4.27E-04 | 0.46 |
| *Blautia*_A *fusiformis* | Thymine | 9.66E-04 | 0.43 |
| *Blautia*_A *fusiformis* | Hypoxanthine | 6.36E-06 | 0.59 |
| *Blautia*_A *fusiformis* | N-acetylspermidine | 1.13E-04 | 0.51 |
| *Blautia*_A *fusiformis* | Bilirubin | 1.96E-04 | -0.49 |
| *Anaerobutyricum hallii* | Imidazole propionate | 1.19E-03 | 0.42 |
| *Anaerobutyricum hallii* | Glutamate | 1.25E-03 | 0.42 |
| *Anaerobutyricum hallii* | N-acetylspermidine | 1.98E-03 | 0.4 |
| *Dorea*_A *sp019421265* | Imidazole propionate | 3.11E-05 | 0.55 |
| *Dorea*_A *sp019421265* | Glutamate | 4.43E-04 | 0.46 |
| *Dorea*_A *sp019421265* | Uracil | 9.66E-04 | 0.43 |
| *Dorea*_A *sp019421265* | Hypoxanthine | 1.25E-04 | 0.5 |
| *Dorea*_A *sp019421265* | N-acetylspermidine | 8.99E-07 | 0.63 |
| *Blautia*_A *faecis* | Adrenic acid | 1.77E-03 | -0.4 |
| *Blautia*_A *faecis* | Lithocholic acid | 5.84E-04 | 0.45 |
| *Blautia*_A *faecis* | Deoxycholic acid | 9.35E-04 | 0.43 |
| *Blautia*_A *faecis* | Imidazole propionate | 1.66E-03 | 0.41 |
| *Blautia*_A *faecis* | Glutamate | 9.66E-04 | 0.43 |
| *Blautia*_A *faecis* | N-acetylhistidine | 6.60E-04 | 0.44 |
| *Blautia*_A *faecis* | Thymine | 7.80E-04 | 0.44 |
| *Blautia*_A *faecis* | Hypoxanthine | 1.46E-03 | 0.41 |
| *Blautia*_A *faecis* | Pseudouridine | 1.16E-03 | 0.42 |
| *Blautia*_A *faecis* | N-acetylspermidine | 1.24E-04 | 0.5 |
| *Blautia*_A *faecis* | Bilirubin | 9.73E-04 | -0.43 |
| *Anaerostipes amylophilus* | Imidazole propionate | 1.96E-04 | 0.49 |
| *Anaerostipes amylophilus* | Glutamate | 5.95E-05 | 0.53 |
| *Anaerostipes amylophilus* | N-acetylhistidine | 1.87E-04 | 0.49 |
| *Anaerostipes amylophilus* | Uracil | 5.20E-05 | 0.53 |
| *Anaerostipes amylophilus* | Thymine | 1.05E-03 | 0.43 |
| *Anaerostipes amylophilus* | Hypoxanthine | 5.95E-05 | 0.53 |
| *Anaerostipes amylophilus* | N-acetylspermidine | 3.49E-05 | 0.55 |
| *Blautia*_A *obeum* | Lithocholic acid | 5.77E-04 | 0.45 |
| *Blautia*_A *obeum* | Imidazole propionate | 8.61E-05 | 0.52 |
| *Blautia*_A *obeum* | Glutamate | 2.22E-04 | 0.48 |
| *Blautia*_A *obeum* | N-acetylhistidine | 1.39E-05 | 0.57 |
| *Blautia*_A *obeum* | Uracil | 1.28E-03 | 0.42 |
| *Blautia*_A *obeum* | Thymine | 2.26E-04 | 0.48 |
| *Blautia*_A *obeum* | Hypoxanthine | 2.20E-04 | 0.48 |
| *Blautia*_A *obeum* | Pseudouridine | 1.65E-03 | 0.41 |
| *Blautia*_A *obeum* | N-acetylspermidine | 5.18E-06 | 0.6 |
| *Blautia*_A *sp000436615* | Lithocholic acid | 3.91E-04 | 0.46 |
| *Blautia*_A *sp000436615* | Imidazole propionate | 6.74E-05 | 0.52 |
| *Blautia*_A *sp000436615* | Glutamate | 1.66E-04 | 0.49 |
| *Blautia*_A *sp000436615* | Creatine | 9.84E-04 | -0.43 |
| *Blautia*_A *sp000436615* | N-acetylhistidine | 1.19E-03 | 0.42 |
| *Blautia*_A *sp000436615* | Thymine | 6.58E-04 | 0.45 |
| *Blautia*_A *sp000436615* | Hypoxanthine | 2.00E-03 | 0.4 |
| *Blautia*_A *sp000436615* | Pseudouridine | 7.45E-04 | 0.44 |
| *Blautia*_A *sp000436615* | N-acetylspermidine | 6.89E-06 | 0.59 |
| *Blautia*_A *sp000436615* | Erythronic acid | 1.27E-03 | -0.41 |
| *Blautia*_A *sp000436615* | Bilirubin | 1.04E-03 | -0.43 |
| *Oliverpabstia faecicola* | N-acetylhistidine | 8.91E-04 | 0.43 |
| *Oliverpabstia faecicola* | Thymine | 1.07E-03 | 0.42 |
| *Oliverpabstia faecicola* | N-acetylspermidine | 1.93E-03 | 0.4 |
| *Oliverpabstia faecicola* | Erythronic acid | 1.77E-03 | -0.4 |
| *Oliverpabstia faecicola* | Bilirubin | 1.89E-04 | -0.49 |
| *Oliverpabstia tarda* | Isovaleric acid | 7.45E-04 | 0.44 |
| *Oliverpabstia tarda* | Caproic acid | 1.66E-03 | 0.41 |
| *Oliverpabstia tarda* | Adrenic acid | 1.25E-04 | -0.5 |
| *Oliverpabstia tarda* | Lithocholic acid | 1.54E-03 | 0.41 |
| *Oliverpabstia tarda* | Imidazole propionate | 1.44E-03 | 0.41 |
| *Oliverpabstia tarda* | Glutamate | 1.07E-03 | 0.43 |
| *Oliverpabstia tarda* | N-acetylhistidine | 1.89E-04 | 0.49 |
| *Oliverpabstia tarda* | Thymine | 5.04E-05 | 0.53 |
| *Oliverpabstia tarda* | N-acetylspermidine | 7.40E-05 | 0.52 |
| *Oliverpabstia tarda* | Erythronic acid | 1.99E-04 | -0.48 |
| *Oliverpabstia tarda* | Bilirubin | 2.20E-04 | -0.48 |
| *Lentihominibacter faecis* | Body mass index | 1.27E-03 | 0.41 |
| *Lentihominibacter faecis* | Waist/hip | 1.24E-03 | 0.42 |
| *Faecalibacillus intestinalis* | Body mass index | 5.95E-05 | 0.52 |
| *Faecalibacillus intestinalis* | Body fat rate | 1.16E-03 | 0.42 |
| *Faecalibacillus intestinalis* | Waist/hip | 1.05E-04 | 0.5 |
| *Faecalibacillus intestinalis* | Basal metabolic rate/fat free mass | 1.54E-04 | -0.5 |
| *Blautia stercoris* | Body mass index | 4.14E-04 | 0.46 |
| *Blautia stercoris* | Muscle mass/weight | 1.77E-03 | -0.41 |
| *Blautia stercoris* | Waist/hip | 6.42E-04 | 0.44 |
| *Dorea formicigenerans* | Body mass index | 1.68E-05 | 0.56 |
| *Dorea formicigenerans* | Body fat rate | 2.56E-04 | 0.47 |
| *Dorea formicigenerans* | Muscle mass/weight | 5.35E-04 | -0.45 |
| *Dorea formicigenerans* | Waist/hip | 2.72E-05 | 0.55 |
| *Dorea formicigenerans* | Basal metabolic rate/fat free mass | 6.04E-04 | -0.45 |
| *Fusicatenibacter saccharivorans* | Basal metabolic rate/fat free mass | 1.33E-03 | -0.42 |
| *Blautia*_A *sp900066335* | Body mass index | 4.07E-05 | 0.53 |
| *Blautia*_A *sp900066335* | Body fat rate | 1.25E-04 | 0.5 |
| *Blautia*_A *sp900066335* | Muscle mass/weight | 6.49E-04 | -0.45 |
| *Blautia*_A *sp900066335* | Waist/hip | 5.04E-05 | 0.53 |
| *Blautia*_A *sp900066335* | Basal metabolic rate/fat free mass | 1.75E-04 | -0.49 |
| *Blautia*_A *wexlerae* | Body mass index | 1.05E-04 | 0.5 |
| *Blautia*_A *wexlerae* | Body fat rate | 7.43E-04 | 0.44 |
| *Blautia*_A *wexlerae* | Muscle mass/weight | 9.66E-04 | -0.43 |
| *Blautia*_A *wexlerae* | Waist/hip | 1.66E-04 | 0.49 |
| *Blautia*_A *wexlerae* | Basal metabolic rate/fat free mass | 5.72E-05 | -0.53 |
| *Anaerobutyricum soehngenii* | Body mass index | 1.21E-04 | 0.5 |
| *Anaerobutyricum soehngenii* | Body fat rate | 6.92E-04 | 0.44 |
| *Anaerobutyricum soehngenii* | Waist/hip | 2.19E-04 | 0.48 |
| *Anaerobutyricum soehngenii* | Basal metabolic rate/fat free mass | 3.05E-04 | -0.47 |
| *Blautia*_A *fusiformis* | Body mass index | 7.99E-04 | 0.43 |
| *Blautia*_A *fusiformis* | Body fat rate | 8.12E-04 | 0.43 |
| *Blautia*_A *fusiformis* | Muscle mass/weight | 7.49E-04 | -0.44 |
| *Blautia*_A *fusiformis* | Basal metabolic rate/fat free mass | 8.15E-04 | -0.44 |
| *Anaerobutyricum hallii* | Body mass index | 7.19E-05 | 0.51 |
| *Anaerobutyricum hallii* | Body fat rate | 1.73E-05 | 0.56 |
| *Anaerobutyricum hallii* | Muscle mass/weight | 2.87E-05 | -0.55 |
| *Anaerobutyricum hallii* | Waist/hip | 5.04E-05 | 0.53 |
| *Anaerobutyricum hallii* | Basal metabolic rate/fat free mass | 1.12E-03 | -0.42 |
| *Dorea*_A *sp019421265* | Body mass index | 9.09E-05 | 0.51 |
| *Dorea*_A *sp019421265* | Body fat rate | 8.15E-04 | 0.43 |
| *Dorea*_A *sp019421265* | Muscle mass/weight | 1.41E-03 | -0.41 |
| *Dorea*_A *sp019421265* | Waist/hip | 2.20E-04 | 0.48 |
| *Dorea*_A *sp019421265* | Basal metabolic rate/fat free mass | 1.20E-04 | -0.51 |
| *Blautia*_A *faecis* | Body mass index | 2.05E-04 | 0.48 |
| *Blautia*_A *faecis* | Body fat rate | 6.47E-05 | 0.52 |
| *Blautia*_A *faecis* | Muscle mass/weight | 1.25E-04 | -0.5 |
| *Blautia*_A *faecis* | Waist/hip | 1.20E-04 | 0.5 |
| *Anaerostipes amylophilus* | Body mass index | 5.04E-05 | 0.53 |
| *Anaerostipes amylophilus* | Body fat rate | 8.78E-04 | 0.43 |
| *Anaerostipes amylophilus* | Waist/hip | 9.07E-05 | 0.51 |
| *Anaerostipes amylophilus* | Basal metabolic rate/fat free mass | 1.65E-03 | -0.41 |
| *Blautia*_A *obeum* | Body mass index | 5.72E-05 | 0.52 |
| *Blautia*_A *obeum* | Body fat rate | 2.97E-04 | 0.47 |
| *Blautia*_A *obeum* | Muscle mass/weight | 8.69E-04 | -0.43 |
| *Blautia*_A *obeum* | Waist/hip | 1.25E-04 | 0.5 |
| *Blautia*_A *obeum* | Basal metabolic rate/fat free mass | 1.28E-03 | -0.42 |
| *Blautia*_A *sp000436615* | Body mass index | 3.47E-04 | 0.46 |
| *Blautia*_A *sp000436615* | Body fat rate | 1.68E-04 | 0.49 |
| *Blautia*_A *sp000436615* | Muscle mass/weight | 2.50E-04 | -0.48 |
| *Blautia*_A *sp000436615* | Waist/hip | 1.80E-04 | 0.49 |
| *Oliverpabstia tarda* | Body mass index | 9.68E-04 | 0.43 |
| *Oliverpabstia tarda* | Waist/hip | 7.49E-04 | 0.44 |
| Inoviridae | p-Cresol synthesis | 2.20E-04 | 0.48 |
| Inoviridae | Urea degradation | 4.27E-04 | 0.46 |
| Inoviridae | Acetate to acetyl-CoA | 6.49E-04 | 0.45 |
| Inoviridae | 4-aminobutyrate degradation | 0.00E+00 | 0.65 |
| Inoviridae | Valine degradation | 2.42E-04 | 0.48 |
| Inoviridae | G-hydroxybutyric acid degradation | 6.47E-05 | 0.52 |
| Inoviridae | Arginine degradation I | 4.43E-04 | 0.46 |
| Myoviridae | p-Cresol synthesis | 0.00E+00 | 0.67 |
| Myoviridae | Aspartate degradation I | 8.56E-06 | 0.58 |
| Myoviridae | Methionine degradation I | 1.56E-03 | 0.41 |
| Myoviridae | Glycine degradation | 2.19E-04 | 0.48 |
| Myoviridae | Urea degradation | 1.76E-06 | 0.62 |
| Myoviridae | Glycerol degradation II | 1.05E-03 | 0.43 |
| Myoviridae | Sulfate reduction (dissimilatory) | 6.03E-04 | 0.45 |
| Myoviridae | Acetate to acetyl-CoA | 2.75E-07 | 0.64 |
| Myoviridae | 4-aminobutyrate degradation | 1.47E-04 | 0.5 |
| Myoviridae | G-hydroxybutyric acid degradation | 0.00E+00 | 0.7 |
| Myoviridae | Arginine degradation I | 6.25E-06 | 0.59 |
| Myoviridae | N-acetylspermidine | 1.46E-03 | 0.41 |
| Myoviridae | Erythronic acid | 3.04E-04 | -0.47 |
| Myoviridae | Bilirubin | 1.66E-03 | -0.41 |
| Myoviridae | Body fat rate | 6.61E-04 | 0.44 |
| Myoviridae | Muscle mass/weight | 1.69E-03 | -0.41 |
| p-Cresol synthesis | Erythronic acid | 1.28E-03 | -0.41 |
| Aspartate degradation I | Lithocholic acid | 1.88E-03 | 0.4 |
| Aspartate degradation I | Deoxycholic acid | 1.73E-03 | 0.41 |
| Aspartate degradation I | N-acetylhistidine | 1.38E-03 | 0.41 |
| Aspartate degradation I | N-acetylspermidine | 6.33E-04 | 0.45 |
| Aspartate degradation I | Bilirubin | 6.57E-04 | -0.45 |
| Glycine degradation | Lithocholic acid | 7.45E-04 | 0.44 |
| Glycine degradation | N-acetylhistidine | 4.89E-04 | 0.46 |
| Glycine degradation | N-acetylspermidine | 4.46E-04 | 0.46 |
| Glycine degradation | Erythronic acid | 9.30E-04 | -0.43 |
| Glycine degradation | Bilirubin | 4.79E-04 | -0.46 |
| Urea degradation | Deoxycholic acid | 1.64E-03 | 0.41 |
| Urea degradation | Bilirubin | 9.99E-04 | -0.43 |
| Glycerol degradation II | N-acetylspermidine | 1.23E-03 | 0.42 |
| Acetate to acetyl-CoA | Adrenic acid | 1.70E-03 | -0.4 |
| Acetate to acetyl-CoA | Erythronic acid | 9.24E-04 | -0.43 |
| 4-aminobutyrate degradation | Adrenic acid | 6.58E-04 | -0.44 |
| 4-aminobutyrate degradation | Lithocholic acid | 7.54E-04 | 0.44 |
| 4-aminobutyrate degradation | Deoxycholic acid | 1.10E-03 | 0.42 |
| 4-aminobutyrate degradation | Creatine | 1.82E-03 | -0.4 |
| 4-aminobutyrate degradation | Thymine | 8.69E-04 | 0.43 |
| 4-aminobutyrate degradation | N-acetylspermidine | 1.09E-03 | 0.42 |
| 4-aminobutyrate degradation | Bilirubin | 1.03E-03 | -0.43 |
| Valine degradation | Lithocholic acid | 2.26E-04 | 0.48 |
| Valine degradation | Deoxycholic acid | 1.44E-04 | 0.5 |
| Valine degradation | Hypoxanthine | 8.33E-04 | 0.44 |
| Valine degradation | N-acetylspermidine | 3.15E-04 | 0.47 |
| G-hydroxybutyric acid degradation | Glutamate | 8.91E-04 | 0.43 |
| G-hydroxybutyric acid degradation | N-acetylhistidine | 1.77E-03 | 0.41 |
| G-hydroxybutyric acid degradation | Thymine | 9.66E-04 | 0.43 |
| G-hydroxybutyric acid degradation | Hypoxanthine | 1.05E-03 | 0.43 |
| G-hydroxybutyric acid degradation | N-acetylspermidine | 5.97E-05 | 0.53 |
| G-hydroxybutyric acid degradation | Bilirubin | 1.37E-03 | -0.42 |
| Arginine degradation I | Caproic acid | 7.43E-04 | 0.44 |
| Arginine degradation I | Adrenic acid | 6.28E-04 | -0.44 |
| Arginine degradation I | Lithocholic acid | 7.26E-04 | 0.44 |
| Arginine degradation I | Creatine | 1.81E-03 | -0.4 |
| Arginine degradation I | N-acetylspermidine | 5.70E-04 | 0.45 |
| Arginine degradation I | Erythronic acid | 7.24E-04 | -0.44 |
| Aspartate degradation I | Body fat rate | 1.65E-03 | 0.41 |
| Methionine degradation I | Body fat rate | 4.63E-04 | 0.45 |
| Methionine degradation I | Muscle mass/weight | 5.59E-04 | -0.45 |
| Glycine degradation | Body mass index | 9.45E-04 | 0.43 |
| Glycine degradation | Body fat rate | 3.80E-04 | 0.46 |
| Glycine degradation | Muscle mass/weight | 6.67E-04 | -0.44 |
| Glycine degradation | Waist/hip | 4.36E-04 | 0.45 |
| Acetate to acetyl-CoA | Body fat rate | 1.96E-04 | 0.48 |
| Acetate to acetyl-CoA | Muscle mass/weight | 4.04E-04 | -0.46 |
| Imidazole propionate | Basal metabolic rate/fat free mass | 1.65E-03 | -0.41 |
| N-acetylhistidine | Body mass index | 4.41E-04 | 0.45 |
| N-acetylhistidine | Waist/hip | 1.02E-03 | 0.42 |
| Uracil | Basal metabolic rate/fat free mass | 1.86E-03 | -0.4 |
| Hypoxanthine | Basal metabolic rate/fat free mass | 1.96E-04 | -0.49 |
| N-acetylspermidine | Body mass index | 2.20E-04 | 0.48 |
| N-acetylspermidine | Waist/hip | 5.59E-04 | 0.45 |
| N-acetylspermidine | Basal metabolic rate/fat free mass | 1.31E-04 | -0.5 |
